# Persistent androgen receptor-mediated transcription in castration-resistant prostate cancer under androgen-deprived conditions

**DOI:** 10.1093/nar/gks888

**Published:** 2012-09-27

**Authors:** Keith F. Decker, Dali Zheng, Yuhong He, Tamara Bowman, John R. Edwards, Li Jia

**Affiliations:** Department of Medicine, Center for Pharmacogenomics, Washington University School of Medicine, St. Louis, MO 63110, USA

## Abstract

The androgen receptor (AR) is a ligand-inducible transcription factor that mediates androgen action in target tissues. Upon ligand binding, the AR binds to thousands of genomic loci and activates a cell-type specific gene program. Prostate cancer growth and progression depend on androgen-induced AR signaling. Treatment of advanced prostate cancer through medical or surgical castration leads to initial response and durable remission, but resistance inevitably develops. In castration-resistant prostate cancer (CRPC), AR activity remains critical for tumor growth despite androgen deprivation. Although previous studies have focused on ligand-dependent AR signaling, in this study we explore AR function under the androgen-deprived conditions characteristic of CRPC. Our data demonstrate that AR persistently occupies a distinct set of genomic loci after androgen deprivation in CRPC. These androgen-independent AR occupied regions have constitutively open chromatin structures that lack the canonical androgen response element and are independent of FoxA1, a transcription factor involved in ligand-dependent AR targeting. Many AR binding events occur at proximal promoters, which can act as enhancers to augment transcriptional activities of other promoters through DNA looping. We further show that androgen-independent AR binding directs a gene expression program in CRPC, which is necessary for the growth of CRPC after androgen withdrawal.

## INTRODUCTION

One of the major challenges in the management of prostate cancer is the treatment of patients who no longer respond to androgen deprivation therapy. Available treatments for androgen deprivation therapy resistant patients have had modest success, with improvements in survival measured in months (1–5). How prostate cancer cells acquire the ability to survive and proliferate after androgen deprivation is not fully understood. Importantly, the failure of androgen deprivation therapy is not accompanied by the loss of the androgen receptor (AR) or AR activity, but rather with restoration of AR activity through a variety of mechanisms including AR amplification and overexpression, AR mutation (mostly in the ligand-binding domain, conferring ligand promiscuity), increased intratumoral androgen synthesis, androgen-independent AR activation by cytokines and growth factors and constitutively active AR splice variants (6–11).

While mounting evidence shows that AR signaling is critical in both androgen-dependent prostate cancer (ADPC) and castration-resistant prostate cancer (CRPC), important differences in AR-mediated transcription have been observed. Gene expression profiling has shown that the androgen-dependent AR expression program characteristic of ADPC is significantly attenuated in CRPC (12,13). To understand how AR functions in ADPC and CRPC, previous studies have mapped genome-wide androgen-dependent AR-occupied regions in ADPC (such as LNCaP cells) and CRPC (LNCaP-derived cells) cells using chromatin immunoprecipitation (ChIP)-based approaches (14–22). This approach has led to identification of CRPC-specific androgen-dependent AR binding events associated with M-phase cell cycle genes (20), suggesting that androgen-induced AR signaling is altered in CRPC cells through reprogramming of androgen-induced AR binding. Androgen-induced AR reprogramming is also observed after downregulation of FoxA1, a pioneer transcription factor involved in AR targeting and frequently mutated in prostate cancer (21,23,24), although the role of FoxA1 in CRPC remains to be determined. Notably, these studies have focused on AR binding events in the presence of androgen [typically after 10 nM 5α-dihydrotestosterone (DHT) treatment], based on the notion that CRPC growth depends on incomplete androgen suppression and continuous ligand-dependent activation of amplified or hypersensitive AR (6,25,26).

Whereas a ligand-dependent AR-mediated gene expression program may play an important role in CRPC, ligand-independent activation of the AR is believed to account for CRPC growth in a subset of patients. Notably, upregulation of PI3K/AKT, MAPK and HER2/neu signaling promotes androgen-independent growth of prostate cancer *in vitro* and *in vivo* (27–29). Androgen-independent AR DNA binding and transcriptional activity can be induced through increased tyrosine phosphorylation and elevated ubiquitination of AR (30,31). Furthermore, expression of constitutively active AR splice variants lacking the ligand-binding domain occurs frequently in CRPC, and is associated with earlier disease recurrence (32–35). Despite this evidence of androgen-independent AR activation, a detailed study of the existence and biological significance of AR binding events under the androgen-deprived conditions has not been reported.

In this study, we used ChIP-sequencing (ChIP-seq) and RNA-sequencing (RNA-seq) to characterize AR binding events in both the presence and absence of androgen in the well-established LNCaP/C4-2B cell culture model. This model shares strong similarities with the clinical progression from androgen-dependence to castration-resistance (36,37). We observed a significant number of androgen-independent AR binding events that differ substantially from classic androgen-dependent occupancies in CRPC C4-2B cells. In androgen-deprived conditions, the AR persistently occupies a set of genomic loci with constitutively open chromatin structures that lack the canonical androgen response element (ARE) and are not directed by FoxA1. We show that androgen-independent AR binding events lead to a distinct gene expression program and drive CRPC cell growth. Taken together with previous studies, these results suggest that both androgen-dependent and -independent AR expression programs are important mechanisms for the survival and growth of CRPC. The relative importance of these two pathways likely depends on cancer stage and tumor microenvironment. Activation of an alternative androgen-independent AR signaling pathway provides one mechanism by which CRPC cells can survive and grow in androgen-deprived conditions.

## MATERIALS AND METHODS

### Cell culture and materials

LNCaP and C4-2B cells were maintained in RPMI 1640 media with 5% fetal bovine serum (FBS) as previously described (38). Antibodies and siRNA reagents used in this study are listed in Supplementary File S1.

### ChIP and ChIP-seq

LNCaP or C4-2B cells were cultured in phenol red-free RPMI 1640 media supplemented with 5% charcoal-stripped FBS (CSS) for 3 days. After treatment with ethanol or DHT (10 nM) for additional 4 h (for LNCaP) or 16 h (for C4-2B), ChIP experiments were performed as described previously (15,39). For the ChIP after FoxA1 knockdown, C4-2B cells were transfected with FoxA1 siRNA (final 15 nM) or non-target siRNA using Lipofectamine RNAiMAX Transfection Reagent and Reverse Transfection Protocol (Invitrogen), and then grown in phenol red-free RPMI 1640 containing 5% CSS for 3 days prior to ChIP. ChIP DNA was analyzed by quantitative polymerase chain reaction (qPCR) using TaqMan or SYBR PCR Master Mix (Applied Biosystems). The primers and probes are listed in Supplementary File S1.

The ChIP-seq libraries were prepared according to the Illumina Protocol (www.illumina.com) with modifications. Briefly, 10 ng of ChIP DNA was end-repaired, ligated to barcoded adaptors, size-selected on agarose gel (300–500 bp) and PCR amplified for 16 cycles using Phusion polymerase (Finnzymes). The libraries were sequenced in the Illumina Genome Analyzer IIx or HiSeq2000 system according to the manufacturer’s instruction. A summary of ChIP-seq experiments is provided in Supplementary File S1.

### ChIP-seq analysis

ChIP-seq reads were mapped to the human genome (hg18) using Bowtie (40). Reads that did not map uniquely were disregarded. SISSRS (41) was used to identify AR binding sites, with input samples used as background and at a *P*-value threshold of 0.01. DBChip was used to merge sites identified by SISSRS into a list of AR binding sites (±125 bp from the peak center) observed in at least one experiment (42). Binding at a given AR site is reported in counts per million (CPM) uniquely mapped reads. Peaks mapping to ribosomal RNA (rRNA) or satellite repeats [based on RepeatMasker 3.2.7 (43)] were disregarded since they cannot be properly mapped due to incomplete annotation. Binding sites with >1 CPM in C4-2B or LNCaP input samples were also disregarded. Differentially bound sites [false discovery rate (FDR) < 0.1] were identified using edgeR (44) following previously described methods (45). Tag-wise dispersion was modeled in edgeR using the generalized linear model (glm) functionality, with ChIP-seq antibody used as a blocking factor and normalization based on the total number of uniquely mapped reads. Genomic location of peaks was determined relative to the nearest Ensembl transcript with a complete annotation. The gene promoter was defined as ±1 kb relative to the transcription start site (TSS). Transfer RNA (tRNA) annotations were based on Repeat Masker and the GtRNAdb (46). In order to visualize nucleosome depletion at AR bindings sites, 9 (0.13%) androgen-dependent AR-occupied regions with outlying histone H3 lysine 9 and 14 acetylation (AcH3) (maximum signal > 0.5 cpm/bp) were removed when computing the average AcH3 signal.

### Motif finding

The MEME suite of analysis tools was used for motif discovery and detection (47). *De novo* motif discovery using MEME was performed within ±125 bp relative to the ChIP-seq peak center using default MEME–ChIP settings (48). AME was used to test for statistically significant over-representation of motifs (49). Known motifs were obtained from the Jaspar core database (50).

### siRNA transfection

C4-2B cells were grown in phenol red-free RPMI 1640 containing 5% CSS for 2 days. Cells were transfected with siRNA duplexes as indicated at a final concentration of 15 nM using Lipofectamine RNAiMAX Transfection Reagent and Forward (or Reverse) Transfection protocol (Invitrogen). After transfection, cells were grown in phenol red-free RPMI 1640 containing 5% CSS for 48 h and then treated with ethanol or DHT (10 nM) for additional 16 h. Total RNA extraction and protein extraction were conducted for further assessment by RNA-seq, qRT–PCR and western blot.

### RNA-seq

RNA-seq was performed as reported previously with modifications (51,52). Briefly, 10 µg of total RNA was oligo (dT) selected using the Dynabeads mRNA purification kit (Invitrogen) or depleted of rRNA using the RiboMinus kit (Invitrogen) and subsequently fragmented using RNA Fragmentation Reagents (Ambion). The fragmented RNA was randomly primed with hexamers and reverse-transcribed using the Just cDNA Double-stranded cDNA Synthesis kit (Stratagene). After second-strand synthesis, the cDNA was end-repaired, ligated to barcoded adaptors, size selected on agarose gel (150–300 bp) and PCR amplified for 14 cycles using Phusion polymerase (Finnzymes). The libraries were sequenced in the Illumina Genome Analyzer IIx or HiSeq2000 system according to the manufacturer’s instruction. A summary of the RNA-seq experiments is provided in Supplementary File S1.

### RNA-seq analysis

RNA-seq reads were mapped to the human genome (hg18) using Tophat (53). Aligned reads were filtered to eliminate reads that mapped to rRNA and RNA repeats (snRNA, scRNA, srpRNA, tRNA and RNA). Htseq-count (54) was used to obtain raw read counts based on Ensembl gene annotations (hg18 v54) using the union method. Genes that mapped to ribosomal and mitochondrial proteins, or did not have at least 5 counts per million (CPM > 5) uniquely mapped reads in at least two samples were filtered prior to differential testing. Ensembl genes lacking a corresponding RefSeq mRNA entry were also eliminated. Differentially expressed genes (FDR = 0.05 and 1.5-fold change) were identified using edgeR (44) with TMM normalization (55) and tag-wise dispersion. Gene ontology analysis was performed using GOstats (56) and MetaCore from GeneGo Inc. Gene set enrichment analysis (57) was performed using the Bioconductor package phenoTest (58), with curated gene signatures obtained from the GeneSigDB (59). Gene expression is reported in CPM or fragments per kilobase of exon per million mapped reads (FPKM).

### qRT–PCR

After the indicated treatments, total RNA from cells was extracted using TRIzol Reagent (Invitrogen). cDNA was prepared through reverse transcription (RT) using the iScript cDNA Synthesis Kit (Bio-Rad), and qPCR was conducted using SYBR Green PCR Master Mix (Applied Biosystems). Triplicate PCR reactions were conducted. glyceraldehyde 3-phosphate dehydrogenase (*GAPDH*) mRNA expression was analyzed for each sample in parallel. The primers are listed in Supplementary File S1.

### Western blot analysis

Western blots were performed as previously described using the indicated antibodies (60).

### Construction of plasmids

In total, 10 androgen-dependent and 10 androgen-independent AR-occupied regions were PCR amplified from C4-2B genomic DNA and subcloned upstream of a minimal promoter into pGL4.26 vector (Promega). Five out of 10 androgen-independent AR-occupied regions are located at the promoter regions, which were cloned in reverse direction to minimize the promoter activity in luciferase assays. Also, 10 random genomic regions were subcloned into pGL4.26 vector and used as controls. The plasmid sequences were confirmed by Sanger sequencing. The primers for cloning are listed in Supplementary File S1.

### Luciferase assay

LNCaP or C4-2B cells (4 × 10^4^ cells/well) were plated in 48-well plates and grown in phenol red-free RPMI 1640 containing 5% CSS for 2 days. Cells were then transfected with luciferase reporter plasmids (100 ng/well) using Lipofectamine LTX Reagent (Invitrogen). pRL-TK renilla luciferase plasmid (10 ng/well) (Promega) was co-transfected as an internal control. For the luciferase assay after AR knockdown, cells were transfected with AR siRNA (final 15 nM) using Lipofectamine RNAiMAX Transfection Reagent and Reverse Transfection Protocol (Invitrogen), and then grown in phenol red-free RPMI 1640 containing 5% CSS for 2 days prior to reporter plasmid transfection. After plasmid transfection, cells were treated with ethanol or DHT (10 nM) for 24 h. Firefly and renilla luciferase activities were measured using the Dual-Glo Luciferase Assay System (Promega). Results are represented as firefly/renilla ratio.

### Cell proliferation and apoptosis assays

C4-2B cells (1 × 10^4^ cells/well) were plated in 96-well plates and transfected with gene-specific siRNA at a final concentration of 15 nM using Lipofectamine RNAiMAX Transfection Reagent and Reverse Transfection Protocol. For proliferation assay, cells were maintained in phenol red-free RPMI 1640 containing 5% CSS with ethanol or different concentrations of R1881 as indicated for 5 days. The synthetic androgen R1881 was used instead of DHT to minimize metabolic degradation during incubation. The number of viable cells was analyzed using the CCK-8 kit (Dojindo Molecular Technologies). For apoptosis assay, cells were grown in phenol red-free RPMI 1640 containing 5% CSS with ethanol or DHT (10 nM) for 3 days after siRNA transfection. The Caspase 3/7 activity was measured using the Caspase-Glo 3/7 Assay kit (Promega).

### Chromatin conformation capture assay

Chromatin conformation capture (3C) assays were performed as previously reported with modifications (61–63). Briefly, LNCaP or C4-2B cells (1 × 10^7^ cells/150 mm dish) were grown in phenol red-free RPMI 1640 containing 5% CSS for 3 days. Cells were fixed with 1% formaldehyde for 10 min at room temperature, and then lysed in cold lysis buffer [10 mM Tris–HCl (pH 7.5), 10 mM NaCl, 0.2% Igepal CA-630, 1 × protease inhibitor cocktail (Roche)]. The nuclei were harvested and suspended in digestion buffer containing 0.3% SDS and 2% Triton X-100. The chromatin was digested with BamHI or EcoRI (New England Biolabs) overnight at 37°C while shaking at 900 rpm. The reaction was then diluted with ligation buffer containing 0.1% SDS and 1% Triton X-100 in a final volume of 7 ml. Ligation was incubated at 16°C overnight with 2000 U T4 DNA ligase (New England Biolabs), followed by overnight incubation at 65°C in the presence of 10 μg/ml proteinase K (Sigma) to reverse cross-linking. The DNA was isolated by phenol–chloroform extraction and ethanol precipitation. The purified DNA was quantified and used as a PCR template.

To create a standard for normalization of different PCR efficiencies, 3C control template was generated by digesting an equimolar mix of the PCR fragments spanning all restriction sites of interest followed by ligation to produce all possible ligation products (62). To control for differences of the 3C efficiency in different samples, the interaction of two sites at the TUBG2 locus was utilized as an internal control. TUBG2 is equally expressed in both cell lines. The efficiency of chromatin digestion at BamHI and EcoRI sites was >80% determined by qPCR amplifying a fragment spanning a BamHI or EcoRI site in the uncut and cut chromatin. A Taqman probe and a forward (or reverse) primer were designed specifically to a BamHI or EcoRI fragment at the AI-OR of interest. Multiple reverse (or forward) primers were then designed, which were specific to different BamHI or EcoRI fragments across the whole region. All qPCR reactions were carried out in duplicate and compared against standard curves of 3C control template. The interaction frequency of the closest point to the AI-OR in C4-2B cells was defined as 100. The results are presented as the mean ± standard deviation of two independent 3C preparations. Sequences for primers and probes are listed in Supplementary File S1.

## RESULTS

### Identification of androgen-independent AR binding events in CRPC cells

**Figure 1.**
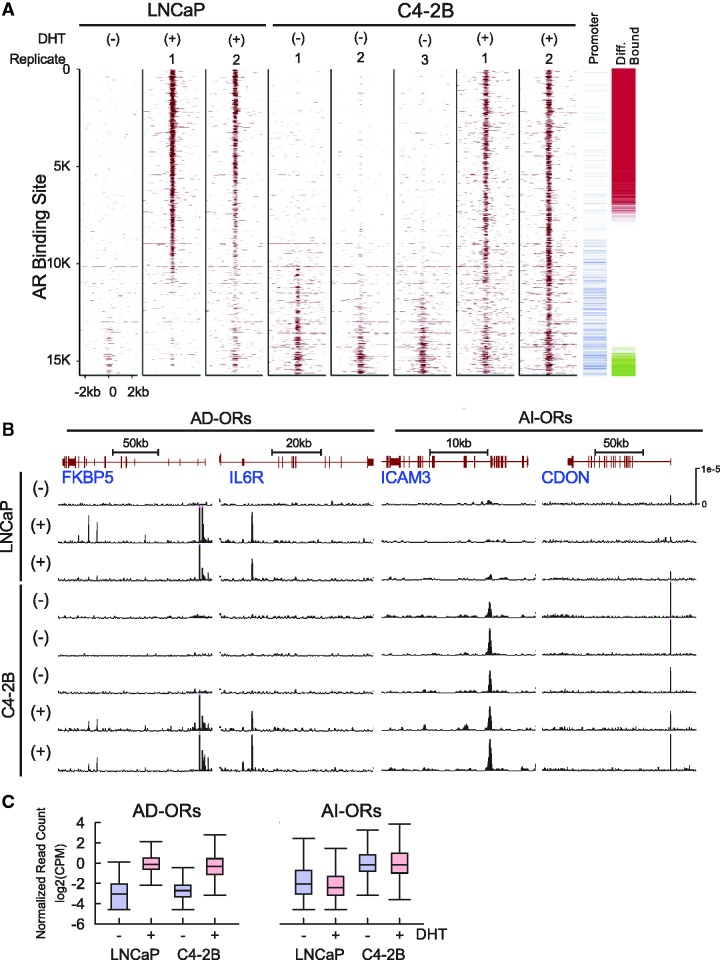
Androgen-independent AR binding in CRPC cells. (**A**) DHT dependence of AR ChIP-seq signal intensity in a ±2 kb region around 15 709 AR binding sites observed in at least 1 ChIP-seq sample in LNCaP DHT−, LNCaP DHT+ (two replicates), C4-2B DHT− (three replicates) and C4-2B DHT+ (two replicates). AR binding sites located within ±1 kb of a TSS are marked in blue (promoter). AR binding sites showing increased binding in LNCaP DHT+ versus C4-2B DHT− are marked with red (androgen-dependent occupied regions or AD-ORs), whereas AR binding sites showing increased binding in C4-2B DHT− versus LNCaP DHT+ are marked green (androgen-independent occupied regions or AI-ORs) (**B**) Normalized ChIP-seq read counts at representative AD-ORs (left) and AI-ORs (right) (**C**) DHT dependence of average AR binding at AD-ORs and AI-ORs in LNCaP and C4-2B cells. Average binding is shown in log2 CPM, normalized by the number of uniquely mapped reads in the sample.

The LNCaP cell line, which expresses a functional albeit mutant AR, has a robust transcriptional response to androgen (38) and depends on androgen for cell proliferation (Supplementary Figure S1A). C4-2B is a CRPC cell line derived from a LNCaP xenograft that relapsed and metastasized to bone after castration. C4-2B cells show similar growth rates in the presence or absence of androgen. In the presence of androgen, C4-2B cell growth is inhibited by the AR antagonist bicalutamide, indicating androgen-dependent AR signaling remains functional (Supplementary Figure S1B and C). In the absence of androgen, however, growth of the C4-2B cells is minimally affected by bicalutamide but strongly inhibited by siRNA against AR (Supplementary Figure S1D and E). These results suggest that C4-2B cells in androgen-deprived conditions exhibit androgen-independent but AR-dependent growth. To understand how AR promotes C4-2B cell growth under androgen-deprived conditions, we asked whether AR genomic binding events in the absence of androgen are present and comparable with classic androgen-dependent binding events. We mapped AR binding sites in LNCaP and C4-2B cells in the presence and absence of DHT (10 nM) using ChIP-seq. We identified a total of 15 709 AR binding events in at least one sample at a *P*-value threshold of 0.01 (Figure 1A). In line with previous studies, a large number of DHT-dependent AR binding sites are observed in both LNCaP and C4-2B cells (16,20,22). Most remarkably, we identified a set of AR binding events persistently present in C4-2B cells even after androgen withdrawal. Differential binding analysis (45) was used to identify AR occupied regions with statistically significant differential binding in C4-2B DHT− versus LNCaP DHT+ cells (FDR = 0.1). We refer to the 7135 AR binding sites with statistically increased binding in LNCaP DHT+ cells as androgen-dependent occupied regions (AD-ORs), whereas we refer to the 896 sites with statistically increased binding in C4-2B DHT− cells as androgen-independent occupied regions (AI-ORs) (AD-OR and AI-OR examples in Figure 1B and full list in Supplementary File S2). Selected AD and AI-ORs were validated by ChIP–qPCR and showed good agreement with ChIP-seq data (Supplementary Figure S2). We hypothesized that AI-ORs are responsible for the castration-resistant, AR-dependent phenotype in C4-2B cells.

We observed similar DHT-dependent occupancy of AD-ORs in LNCaP and C4-2B cells (Figure 1C), suggesting that the androgen-dependent AR-mediated expression program remains largely intact in CRPC. The occupancy of AI-ORs in C4-2B cells was globally unaffected by DHT treatment, and in specific cases, decreased (Supplementary Figure S2). Interestingly, we also observed weak occupancy at AI-ORs in parental LNCaP cells (Figure 1A and B), consistent with the hypothesis that C4-2B cells are a selected subpopulation of LNCaP cells (64). We observed a similar pattern of androgen-dependent and androgen-independent AR occupancies in an additional CRPC cell line (22RV1), implying that androgen-independent AR binding is not limited to the C4-2B model (Supplementary Figure S3). The 22RV1 line was derived from a CWR22 xenograft that relapsed during androgen ablation (65). This cell line abundantly expresses a common AR splice variant, which lacks the ligand-binding domain. This truncated protein is constitutively active and frequently detected in CRPC tumors (32–34). Although AR binding in 22RV1 cells is relatively weak compared to C4-2B cells, both common and cell-type specific AR binding events were observed. Whether androgen-independent AR binding in 22RV1 cells is attributable to the AR splice variant lacking the ligand-binding domain remains to be determined.

### AI-ORs possess distinct genomic features from AD-ORs

**Figure 2.**
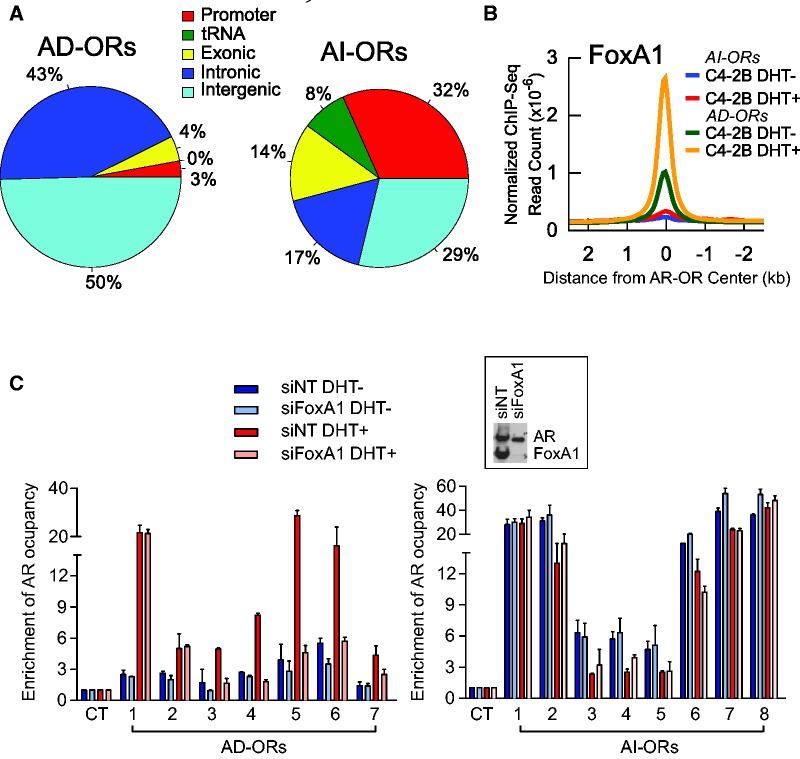
Genomic localization and FoxA1 independence of AR binding at AI-ORs. (**A**) Genomic localization of AD-ORs and AI-ORs. (**B**) DHT-treated and -untreated FoxA1 ChIP-seq signals at AD-ORs and AI-ORs. (**C**) AR occupancies measured by ChIP–qPCR at seven selected AD-ORs and eight AI-ORs after FoxA1 knockdown. AR occupancy at the negative control (CT) region was defined as 1. Knockdown of FoxA1 does not affect AR expression as demonstrated by the western blot (inset). Non-target siRNA (siNT) was used as a control.

We next investigated the properties of 7135 AD-ORs and 896 AI-ORs in C4-2B cells. Whereas the vast majority of AD-ORs are located at intergenic and intronic regions in line with previous findings (15,19), ∼54% of AI-ORs are at promoters, exons and tRNA genes (Figure 2A). Notably, the AR-bound promoter regions were among the strongest AI-ORs (Figure 1A), suggestive of a potential importance in androgen-independent gene regulation.

FoxA1 has been characterized as a pioneer factor involved in chromatin remodeling and facilitation of androgen-dependent AR recruitment. FoxA1 is critical for activation of androgen-dependent transcription (15,66), and downregulation triggers dramatic reprogramming of AR binding (21,23). We next investigated whether FoxA1 plays a similar role in androgen-independent AR binding. Motif analysis showed that both canonical ARE and FoxA1 motifs are not enriched at AI-ORs (Supplementary Figure S4). Although no known motifs were enriched at AI-ORs, we identified a novel motif overrepresented at promoter AI-ORs compared with unbound promoters with no known match in the JASPAR (50), TRANSFAC (67) and UNIPROBE (68) databases. As expected, tRNA A- and B-box motifs are highly enriched at tRNA AI-ORs.

ChIP-seq analysis of genome-wide FoxA1 binding sites in C4-2B cells further revealed that FoxA1 was located at AD-ORs, but not at AI-ORs (Figure 2B and Supplementary Figure S5). Pre-existing FoxA1 binding at AD-ORs was substantially enhanced after DHT treatment in line with previous studies (15), suggesting a role in androgen-mediated transcription other than opening of nucleosomes. We next examined AR occupancies at AD-ORs and AI-ORs using ChIP–qPCR after FoxA1 knockdown. While AR binding at five out of seven AD-ORs was diminished by knockdown of FoxA1 in agreement with FoxA1-directed AR reprogramming (21,23), all eight AR occupancies at AI-ORs remained unchanged (Figure 2C). These results demonstrate that AI-ORs are FoxA1-independent and distinct from classic AD-ORs.

### AI-ORs are preferentially located at genomic loci with constitutively open chromatin structures

**Figure 3.**
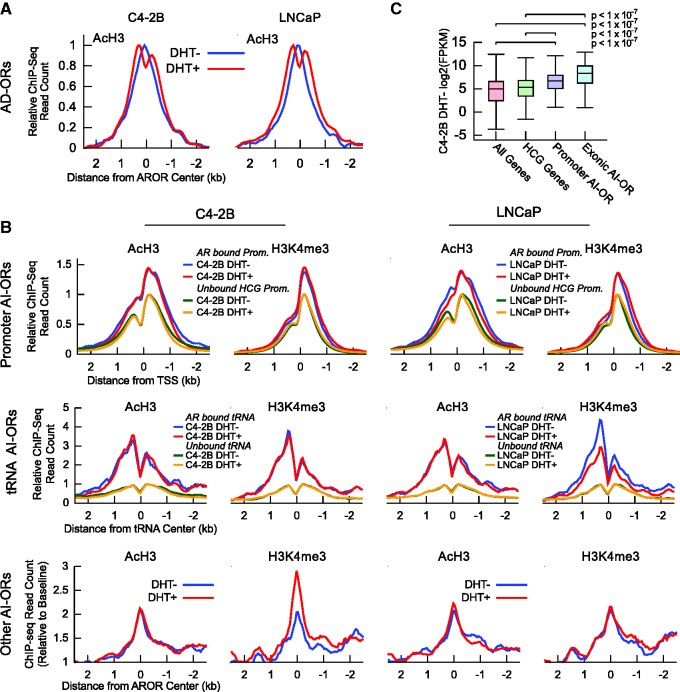
AI-ORs are preferentially located at open chromatin. (**A**) DHT dependence of AcH3 at AD-ORs. Data are plotted as reads per base pair per peak, normalized to the maximum average signal for each experiment. (**B**) (top) DHT dependence of AcH3 and H3K4me3 at 276 AI-OR bound promoters. Data are plotted as reads per base pair per peak, normalized to the maximum average signal at unbound HCG promoters for each experiment. Note that 284 AI-ORs mapped to 276 promoters because some promoters were bound by multiple AI-ORs; (middle) AcH3 and H3K4me3 signal at 54 AI-OR bound and 578 unbound tRNA. AI-OR bound tRNA show a DHT-independent increase in AcH3 and H3K4me3 signal relative to unbound tRNA. Data are plotted as reads per base pair per peak, normalized to the maximum average signal at unbound tRNA for each experiment. Genomic locations of tRNA genes and pseudo-genes were obtained from the Genomic tRNA Database (46). Note that 20 tRNA genes annotated in repeat masker 3.2.7 but not included in the tRNA database were excluded when calculating the average signal; (bottom) AcH3 and H3K4me3 signal at 538 non-promoter and non-tRNA AI-ORs. Bound sites show a DHT-independent increase in signal relative to baseline, defined as the average signal in a region 2.5–5 kb away from the AI-OR center. (**C**) Comparison of expression levels between genes with promoter-bound AI-ORs, exonic AI-ORs, HCG promoters and all genes. Empirical *P*-values were determined by calculating median FPKMs for 1 x 10^7^ simulated trials.

Since AI-ORs lack pre-existing FoxA1 binding, we next asked whether AI-ORs have a unique FoxA1-independent chromatin structure. Histone H3 lysine 9 and 14 acetylation (AcH3) is associated with both promoters and enhancers and frequently marks active AR enhancers (15,69). Upon DHT stimulation, AcH3 signals decreased at the central position of AD-ORs and increased in the flanking regions in both LNCaP and C4-2B cells (Figure 3A). This is indicative of DHT-dependent nucleosome repositioning, which is hypothesized to increase chromatin accessibility and facilitate transcription factor recruitment (70). Since chromatin modification signals vary at different genomic elements, we separated AI-ORs into three categories (promoter, tRNA and other). AI-ORs at AR-bound promoter sites (Figure 3B, top panel) showed strong AcH3 and promoter-specific histone H3 lysine 4 trimethylation (H3K4me3) signals that were unaffected by DHT. Instead, a well defined nucleosome-free region immediately upstream of the TSS was present before and after DHT treatment (Figure 3B). AI-OR binding at promoters most commonly occurred immediately upstream of the TSS near this nucleosome-free region (Supplementary Figure S6A). AR-bound promoters had high CpG (HCG) content (Supplementary Figure S6B) and displayed increased levels of AcH3 and H3K4me3 relative to unbound HCG promoters. AI-ORs at tRNA genes (Figure 3B, middle panel) had a similar chromatin structure to those at promoters, whereas other AI-ORs (Figure 3B, bottom panel) showed elevated AcH3 and H3K4me3 marks centered at the AR binding sites. The lack of a bimodal distribution at the non-promoter/non-tRNA AR binding sites may suggest a distinct nucleosome architecture similar to that of the ‘gained’ AR binding sites observed after FoxA1 knockdown (21). Importantly, these histone modification marks are largely unaffected by DHT treatment. Notably, LNCaP chromatin structure at AI-ORs was similar to that in C4-2B cells (Figure 3B, right). This indicates that whereas open chromatin structures may be required for androgen-independent AR binding, C4-2B AI-OR binding is likely determined by AR DNA binding capacity and AR co-factor activity. The *de novo* promoter motif may also play a role in AR recruitment to specific promoters. In agreement with highly activated epigenetic states, genes associated with AR-bound promoter and exons were expressed at a higher level than unbound genes (Figure 3C).

Collectively, AI-ORs occur at locations with open chromatin structures such as HCG promoters associated with highly expressed genes and other open chromatin regions. The chromatin structure of these regions does not change upon DHT treatment and is independent of FoxA1 binding. These data are consistent with a model where in C4-2B cells a subset of genomic loci with pre-existing accessible chromatin serve as anchoring sites for androgen-independent binding of activated AR.

### AI-ORs possess AR-dependent enhancer activity in CRPC cells

**Figure 4.**
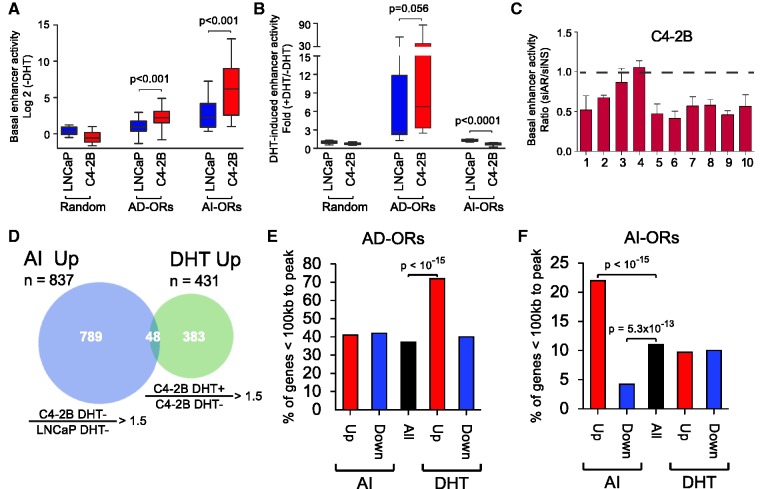
AI-ORs possess AR-dependent enhancer activity and correlate with AI-upregulated genes. (**A**) Luciferase reporter assays showing basal enhancer activity of random regions (*n* = 10), AD-ORs (*n* = 10) and AI-ORs (*n* = 10) in the absence of DHT. *P*-values were calculated based on matched two-tailed Student’s *t*-test. (**B**) DHT-induced enhancer activity of the same regions. (**C**) Basal enhancer activity of AI-ORs after AR knockdown. The data were normalized to non-specific siRNA. (**D**) RNA-seq Venn diagram showing overlap between genes upregulated in C4-2B DHT− versus LNCaP DHT− (termed AI-upregulated) and C4-2B DHT+ versus C4-2B DHT− (termed DHT-upregulated). (**E, F**) Percentage of AI-up and -downregulated, DHT up- and downregulated and all genes within 100 kb of (E) AD-ORs and (F) AI-ORs. *P*-values were calculated based on a hypergeometric test.

We next sought to determine whether AI-ORs exhibited enhancer activity. We examined 10 AD-ORs and 10 AI-ORs in the context of a minimal promoter upstream of the luciferase gene in a transient transfection system. Both AD-ORs and AI-ORs displayed weak basal enhancer activity in LNCaP cells under androgen-deprived conditions compared with randomly selected genomic regions (Figure 4A). We observed higher basal activity at AD-ORs in C4-2B cells compared with that in LNCaP cells likely due to increased sensitivity of C4-2B cells to residual androgens (38). Conversely, remarkably elevated basal activity was observed at AI-ORs in untreated C4-2B cells. As expected, AD-ORs showed DHT-induced enhancer activity in both cell lines (Figure 4B). DHT treatment did not affect enhancer activity of AI-ORs in LNCaP cells, with a fold induction (DHT+/DHT−) of ∼1. In contrast, addition of DHT significantly inhibited enhancer activity at AI-ORs in C4-2B cells. Because AR binding at AI-ORs is not altered by DHT treatment, the decreased enhancer activity is likely due to transcription squelching caused by robust DHT-mediated transcription competing for common AR co-factors. Knockdown of AR resulted in a decrease of basal enhancer activity at 9 out of 10 AI-ORs in C4-2B cells, suggesting that increased DHT-independent enhancer activity depends on AR binding (Figure 4C). This AR-dependent but DHT-independent enhancer activity suggests that AI-ORs may be important regulators of gene expression in the CRPC phenotype.

### AI-ORs regulate a distinct set of distal genes independent of androgen

In order to identify potential targets of AI-OR-mediated gene expression, we next used RNA-seq to identify genes regulated by AR in the presence or absence of DHT and after AR RNA interference (Supplementary Figure S7A). We identified 431 DHT-upregulated genes in C4-2B cells (Figure 4D and full list in Supplementary File S2). In agreement with previous studies (15,19,20), these genes were strongly correlated with AD-ORs based on the proximity of activated genes (Figure 4E). We also identified 837 genes that were upregulated in the absence of DHT in C4-2B compared with LNCaP cells and could potentially account for androgen-independent growth of C4-2B cells. These genes, which we refer to as ‘androgen-independent (AI) upregulated genes’, were largely distinct from DHT-upregulated genes (Figure 4D, genes listed in Supplementary File S2). AI-upregulated genes showed strong genome-wide correlation with AI-ORs but not AD-ORs. Because genome-wide analysis identified a significant number of AI-ORs localized to promoters, we also asked whether AI-OR binding at the proximal promoter correlated with expression of the bound gene. Surprisingly, genes with AI-ORs at the proximal promoter did not show statistically significant upregulation in C4-2B DHT− versus LNCaP DHT− cells (Supplementary Figure S8). These results suggest that promoter bound AI-ORs do not regulate the proximal gene, but instead, regulate gene expression through long-range interactions. The constitutively high expression and open chromatin structure of AI-OR bound promoters likely explains the absence of regulation of the proximal gene.

AI-upregulated genes have a significantly increased probability of downregulation after AR RNA interference (Supplementary Figure S9A), providing further evidence that AR regulates the expression of these genes. Interestingly, AI-upregulated genes also have a significantly increased probability of downregulation after DHT treatment (Supplementary Figure S9B), in line with the reduced enhancer activity of AI-ORs observed in luciferase assays (Figure 4B). Our data thus suggest that a distinct androgen-independent AR regulated gene expression program is active in CRPC cells and is regulated by androgen-independent AR binding. Upon induction of CRPC cells by androgen, this androgen-independent expression program is downregulated and the classic androgen-dependent expression program predominates.

### AI-ORs directly interact with AI-upregulated genes

**Figure 5.**
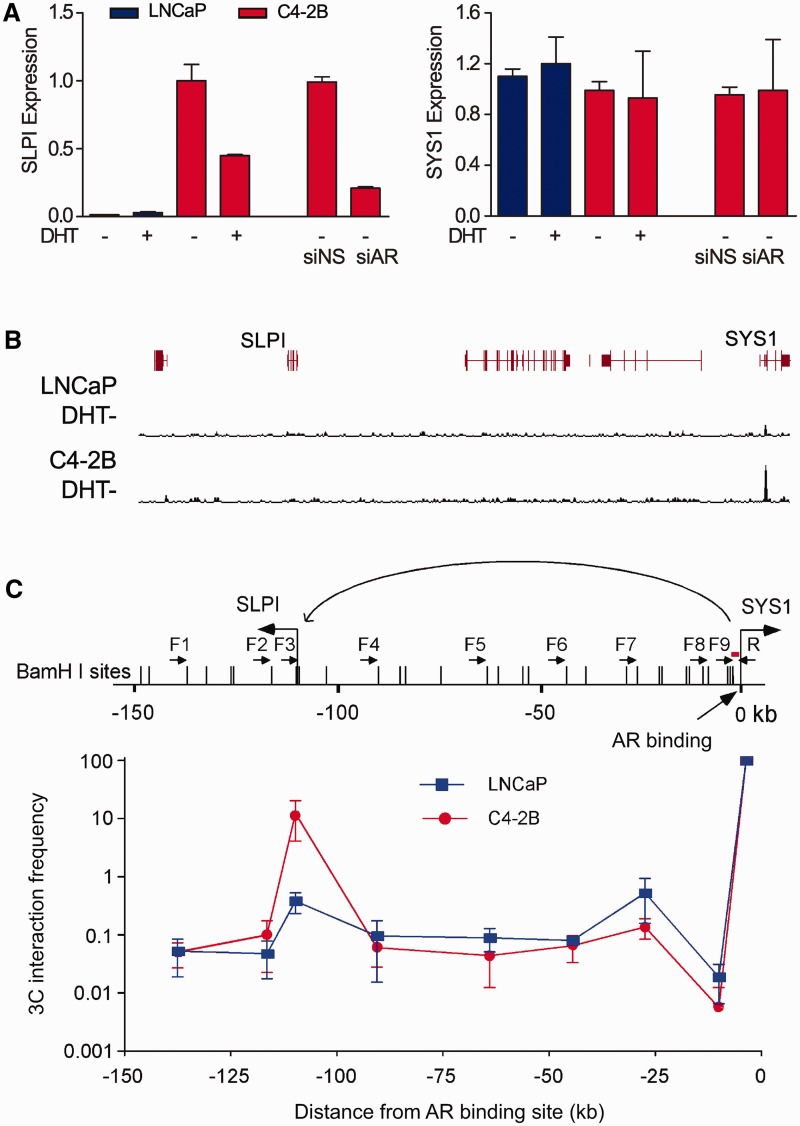
AI-upregulated genes are regulated by distal AI-ORs. (**A**) Real-time RT–PCR results showing expression of *SLPI* and *SYS1* genes in LNCaP and C4-2B cells. *SLPI* but not *SYS1* was downregulated after AR knockdown. (**B**) Normalized ChIP-seq read counts in the region near the AI-OR and the *SYS1* promoter in LNCaP and C4-2B cells. (**C**) 3C assays showing the interaction between the AI-OR at the *SYS1* promoter and the *SLPI* gene promoter. The results are presented as the mean ± standard deviation of two independent 3C preparations. Small black arrows represent the primers and a short red line indicates the probe for 3C-qPCR.

We next sought to confirm the physical interaction between AI-ORs and the distal AI-upregulated genes using the quantitative 3C assay. Our results suggest that AR promoter binding does not regulate the proximal gene, but rather exhibits distal enhancer function. Here, we examined three AI-ORs, two of which were located at promoters. For example, AR was strongly bound to the promoter of the *SYS1* gene in C4-2B cells in the absence of DHT. *SYS1* expression levels were similar between LNCaP and C4-2B cells, and remained unchanged after AR knockdown (Figure 5A), suggesting that direct regulation of this gene by AR was unlikely. In contrast, an AI-upregulated gene, secretory leukocyte peptidase inhibitor (*SLPI*), is located ∼110 kb away from this *SYS1* flanking AI-OR (Figure 5B) and is downregulated by both AR knockdown and DHT treatment. We found that the interaction frequency between the *SYS1* and *SLPI* promoters was significantly increased, compared with nearby regions (Figure 5C). Interestingly, the same interaction was weakly evident in LNCaP cells, consistent with the weak AR binding at AI-ORs observed in LNCaP cells. A similar interaction was demonstrated between another promoter AI-OR and AI-upregulated gene *SERPINH1* (Supplementary Figure S10A). AR-mediated regulation of gene expression through promoter–promoter interactions is consistent with the observation that promoters can exhibit enhancer function and augment the transcriptional activity of other promoters through DNA looping (71). In addition, the interaction between an intergenic AI-OR and nearest AI-upregulated gene *SDC1* was also confirmed by the 3C assay (Suplementary Figure S10B). These results provide direct evidence that AI-ORs interact with the promoters of nearby genes that exhibit increased expression in androgen-deprived CRPC cells. Androgen-independent AR binding likely directly contributes to the androgen-independent AR-regulated expression program found in CRPC.

### AI-upregulated genes are required for CRPC growth

**Figure 6.**
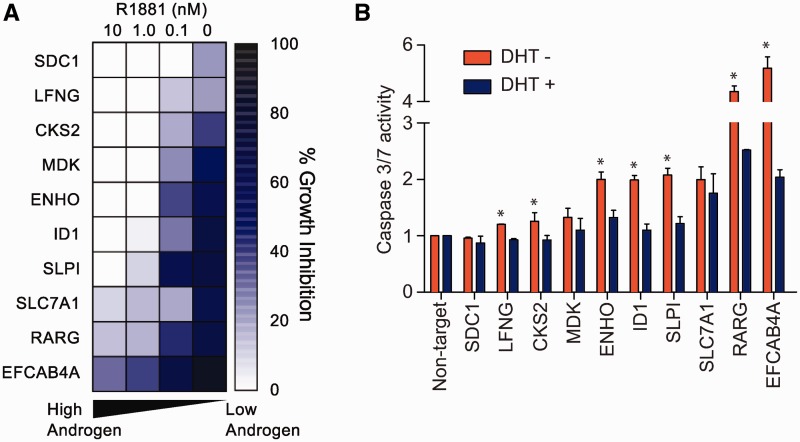
AI-upregulated genes are necessary for CRPC growth. (**A**) Heatmap showing inhibitory effects on C4-2B proliferation 5 days after gene-specific siRNA transfection in different concentrations of R1881. Percentage inhibition is normalized to non-target siRNA. (**B**) Apoptosis assay with C4-2B cells showing Caspase 3 and 7 activity 3 days after gene-specific siRNA transfection in the presence or absence of DHT (10 nM). The results are presented as the mean ± standard deviation of two independent experiments. *P < 0.05, two-tailed Student’s *t*-test.

We next investigated whether AI-upregulated genes are necessary for the survival and proliferation of CRPC cells after androgen withdrawal. We selected 10 AI-upregulated genes for functional analyses, all of which have an androgen-independent AR binding site within 150 kb and are downregulated after AR knockdown. Significant inhibitory effects on C4-2B proliferation after gene-specific RNA interference was observed in the absence of or at low concentrations of androgen (0.1 nM R1881), accompanied by a corresponding increase in apoptosis as determined by caspase-3 and -7 activities (Figure 6). Notably, the inhibition of C4-2B cell proliferation was gradually abrogated when the androgen concentration was increased, presumably due to reactivation of DHT-responsive genes and attenuation of the AI-OR-regulated gene program. These results suggest that androgen-dependent and -independent AR signaling pathways can coexist, but the androgen-independent pathway predominates in the androgen-deprived conditions characteristic of CRPC.

### AI-upregulated genes are enriched for cell cycle functions and overexpressed in CRPC tumors

**Figure 7.**
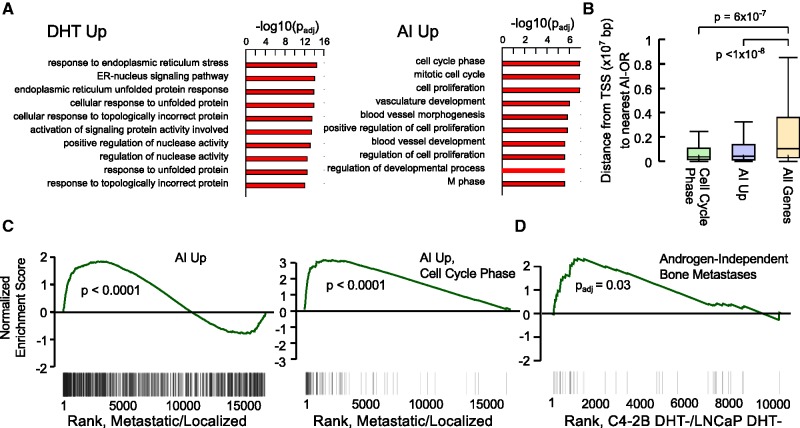
AI-upregulated genes are associated with mitotic cell cycle, proliferation and metastasis. (**A**) Top 10 ontologies enriched in AI-upregulated and DHT-upregulated genes as determined by GOstats (56). *P*-values were calculated based on a hypergeometric test with Benjamini–Hochberg correction. (**B**) Distance from the TSS to the nearest AI-OR for AI-upregulated ‘cell cycle phase’ genes, AI-upregulated genes and all genes. Empirical *P*-values are based on computing the median distance to the nearest AI-OR for randomly selected gene sets using 10^8^ trials. (**C**) AI-upregulated (left) and AI-upregulated ‘cell cycle phase’ genes (right) are enriched in metastatic versus localized CRPC. Genes were ranked by fold change in publicly available metastatic versus localized prostate cancer samples (GSE3325). Black lines denote the position of the AI-upregulated genes within the ranked list. **(D**) A signature of androgen-independent bone metastases (73) is associated with increased expression in C4-2B DHT− versus LNCaP DHT− cells based on gene set enrichment analysis (GSEA). Genes were ranked by fold change in C4-2B DHT− versus LNCaP DHT− cells. Black lines denote position of genes in the androgen-independent bone metastases signature within the ranked list.

We next performed gene ontology and gene set enrichment analysis (GSEA) (57) on DHT and AI-upregulated genes. Whereas DHT-upregulated genes were associated with responses to endoplasmic reticulum stress and protein folding, AI-upregulated genes were highly enriched for cell cycle (M phase), cell proliferation and angiogenesis functions (Figure 7A) as determined using GOstats (56). Enrichment of cell cycle genes was confirmed using an additional analysis tool (MetaCore from GeneGo Inc., Supplementary Figure S11). Notably, AI-upregulated genes involved in cell cycle showed a strong spatial correlation with AI-ORs (Figure 7B). GSEA using a publicly available prostate cancer data set [GSE3325 (72)] showed that both AI-upregulated genes and AI-upregulated ‘cell cycle phase’ genes are significantly upregulated in metastatic prostate tumors (Figure 7C). In addition, GSEA analysis using a database of publicly available gene expression signatures (59) revealed that genes upregulated in C4-2B DHT− versus LNCaP DHT− cells were strongly associated with a signature of CRPC bone metastases (73) (Figure 7D).

The enrichment of mitotic cell cycle genes is consistent with previously reported ontology analysis of genes upregulated in the LNCaP-abl model of CRPC (20). We find significant similarity in gene expression and ontology in the two CRPC models, with 36% of AI-upregulated genes and 69% of AI-upregulated ‘cell cycle phase’ genes also upregulated in LNCaP-abl cells in the absence of androgen (Supplementary Figure S12A), suggesting that similar pathways are activated in response to androgen-deprivation in different models of CRPC. It is important to note, however, that upregulation of LNCaP-abl genes was attributed to DHT-induced AR occupancies, in contrast to the androgen-independent occupancies identified here. Whereas we observed considerable overlap of AD-ORs between C4-2B and LNCaP-abl cells, AI-ORs were largely unique to C4-2B cells (Supplementary Figure S12B). These results suggest that the growth of CRPC can be driven by similar gene expression programs that can be upregulated through different transcriptional mechanisms. These commonly upregulated genes and pathways provide potential therapeutic targets for CRPC treatments against both androgen-dependent and androgen-independent AR signaling.

## DISCUSSION

Given the importance of AR signaling in CRPC, there has been a dedicated interest in dissecting the mechanisms of AR function after androgen deprivation. Many lines of evidence suggest that androgen-dependent AR signaling remains functional in CRPC. It is known that the serum in clinical CRPC is never totally androgen free, that residual androgens are present within the prostate at levels capable of activating the AR despite castration and that enhanced intratumoral androgen synthesis has been commonly observed in CRPC (25,26,74). Furthermore, >50% of CRPC patients showing disease progression on initial lines of hormonal therapies remain responsive to further hormone manipulation (75), suggesting that androgen-dependent AR function remains in CRPC. As a result, AR activity in CRPC has been assessed largely based on androgen-responsive reporters or prostate specific androgen (PSA) production. Next-generation drugs have targeted androgen-dependent AR signaling by inhibition of androgen synthesis (abiraterone) and block of AR ligand-binding (MDV3100) (5,76,77). However, the heterogeneous and often transient response to these new anti-androgen therapies raises the question of whether and how AR-mediated gene transcription occurs in the absence of ligand binding.

Prostate cancer is a molecularly heterogeneous disease even within a single patient, and multiple mechanisms may co-ordinately contribute to CRPC progression. While ligand-dependent AR signaling continues to play an important role in the early stages of CRPC when residual androgen-mediated AR signaling is active, ligand-independent activation of AR may occur in an environment where androgen levels are below castrate levels following severe ligand-depriving therapies. Such therapies have been associated with complete elimination of testosterone in the tumor microenvironment (such as bone) and in some cases a loss of CYP17 (a key enzyme in androgen biosynthesis) in prostate cancer cells (78–80). More importantly, the fact that all anti-androgen approaches eventually fail strongly demonstrates the need to identify and target alternative androgen-independent AR signaling pathways. We reason that androgen-dependent and androgen-independent AR signaling can coexist, and that the relative importance of these two pathways depends on local androgen levels, AR expression and other cellular contexts such as co-regulators (8,81). The androgen-independent AR binding described here occurs at extremely low levels of androgen, which may provide a mechanism for CRPC to develop and survive in a truly androgen-free milieu.

Previous studies have identified AR binding events in the presence of androgen in CRPC cells (15,20,82,83). In this study, we performed AR ChIP-seq in CRPC cells cultured in hormone-depleted media and identified a large number of robust androgen-independent AR binding events. Taken together, these results show that both androgen-dependent and -independent AR signaling play a role in CRPC. The identification of androgen-independent AR binding events does not diminish the importance of androgen-dependent AR signaling. In fact, the androgen-dependent pathway is still intact in CRPC cells and can be rapidly reactivated by androgen stimulation. The fact that androgen-dependent CRPC growth can be inhibited by blocking ligand binding using an AR antagonist further supports the role of androgen-dependent AR signaling in CRPC. In the absence of ligand, however, the AR is no longer directed to canonical AD-ORs, but persistently occupies genomic loci characterized by a pre-existing accessible chromatin structure. These open chromatin structures are often associated with constitutively active genes whose expression is unaffected by AR binding. Instead, AI-ORs interact with neighboring genes and regulate their expression through DNA looping. Androgen-independent AR binding activates a distinct set of cell-cycle genes that can drive cancer cell proliferation after androgen depletion. Although androgen stimulation does not diminish AR occupancies at AI-ORs, expression of AI-OR-associated genes may decrease, likely due to transcription squelching. Inhibition of androgen-independent pathways is accompanied by activation of androgen-dependent pathways, enabling cancer cell survival in the absence or presence of androgen.

Recent studies show that promoter–promoter interactions are widespread in human cells (71,84), with many chromatin complexes spanning 150–200 kb. Our results suggest that AR-bound promoters interact with distal genes through a similar promoter-centered interaction. The AR may function as a bridge between two promoters and bring general transcription machinery from a highly active promoter to a distal target gene. An important question is how the AR is recruited to AI-ORs independent of androgen stimulation. Previous studies showed that AR protein is more active and stable in LNCaP-derived CRPC cells (C4-2 or C4-2B cells) compared with parental cells (38,85,86). AR in C4-2B cells is also predominately localized to the nucleus, suggestive of intrinsic transcriptional activity. There is a growing body of evidence suggesting that the AR can be activated through a range of post-translational modifications (87), which may provide an explanation for higher AR activity and ligand-independent DNA binding in C4-2B cells.

We conclude that androgen-dependent and androgen-independent AR signaling can coexist in CRPC, with their relative importance dependent on AR activity and androgen levels in tumor microenvironment. Androgen deprivation results in a dramatic alteration of genome-wide AR occupancies and reprogramming of AR-mediated gene expression. The androgen-independent AR signaling described here may be an important therapeutic target when androgen-deprivation therapy and anti-androgen treatments fail. More importantly, these results suggest a general mechanism whereby, hormone deprivation reprograms genome-wide hormone receptor binding and gene regulation.

## ACCESSION NUMBERS

RNA-seq and ChIP-seq data have been deposited in the Gene Expression Omnibus (GEO) database (www.ncbi.nlm.nih.gov/geo) under accession number GSE40050.

## SUPPLEMENTARY DATA

Supplementary Data are available at NAR Online: Supplementary Figures 1–12, Supplementary Files 1–3 and Supplementary References [88–90].

## FUNDING

The Concern Foundation; Wendy Will Case Cancer Fund; the Siteman Cancer Center Developmental Research Award in Prostate Cancer Research. Funding for open access charge: The Internal Fund from the Department of Medicine, Washington University in St. Louis.

*Conflict of interest statement*. None declared.

## Supplementary Material

Supplementary Data
