# Supplementary material for: Persistent androgen receptor-mediated transcription in castration-resistant prostate cancer under androgen-deprived conditions
Source: Nucleic Acids Res. 2012 Sep 27;40(21):10765–79. doi: 10.1093/nar/gks888 (PMC3510497; doi:10.1093/nar/gks888)
Supplement: Supplementary Data [file supp_40_21_10765__index.html]

Persistent androgen receptor-mediated transcription in castration-resistant prostate cancer under androgen-deprived conditions — Persistent androgen receptor-mediated transcription in castration-resistant prostate cancer under androgen-deprived conditions — Supplementary Data 

# Persistent androgen receptor-mediated transcription in castration-resistant prostate cancer under androgen-deprived conditions

## Supplementary Data

files

**Files in this Data Supplement:**

- Supplementary Data - pdf file
- Supplementary Data - xlsx file
- Supplementary Data - xlsx file
- Supplementary Data - xlsx file
