# Supplementary material for: Persistent androgen receptor-mediated transcription in castration-resistant prostate cancer under androgen-deprived conditions
Source: Nucleic Acids Res. 2012 Sep 27;40(21):10765–79. doi: 10.1093/nar/gks888 (PMC3510497; doi:10.1093/nar/gks888)
Supplement: Supplementary Data [file supp_gks888_nar-02158-v-2012-File009.pdf]

Supplementary Figures S1-S12

Figure S1. Growth of LNCaP and C4-2B cells in the presence and absence of androgen and androgen receptor (AR). (A) LNCaP or C4-2B cells were grown in phenol red-free RPMI 1640 media with 5% charcoal-stripped serum (CSS) containing ethanol (DHT-) or 1 nM DHT (DHT+). Cells were seeded in 96 well plates (5,000 cells/well) on day 0. Cell proliferation was examined on day 2, 4, and 6 using CCK8 assay kit. **(B)** LNCaP and C4-2B cells were grown in the media as in (A) or in regular RPMI 1640 media with 5% fetal bovine serum (FBS). Bicalutamide (Bic, 10 uM) or DMSO was added on day 0. Cell proliferation was examined using CCK8 assay kit after incubation for 5 days. P-values were calculated based on two-tailed Student's t-test. **(C)** LNCaP (10,000 cells/100 mm dish) and C4-2B (2,000 cells/100 mm dish) cells were seeded in regular RPMI 1640 media with 5% FBS containing bicalutamide (10 uM) or DMSO and incubated for 14 days. Cell colonies were stained using crystal violet. **(D)** C4-2B cells were in phenol red-free RPMI 1640 media with 5% CSS and transfected with 2 different siRNA against AR (siAR, 15 nM). Cell proliferation was examined 5 days after siRNA transfection. P-values were calculated based on two-tailed Student's t-test. High AR knockdown efficiency was confirmed by Western blot. **(E)** C4-2B cells were infected with lentiviruses encoding shRNA against GFP (shGFP) or AR (shAR). At 24h post-infection, cells were grown in the presence or absence of 1 nM R1881 with puromycin selection for 14 days. Cell colonies were stained using crystal violet. High AR knockdown efficiency was confirmed by RT-qPCR.

A

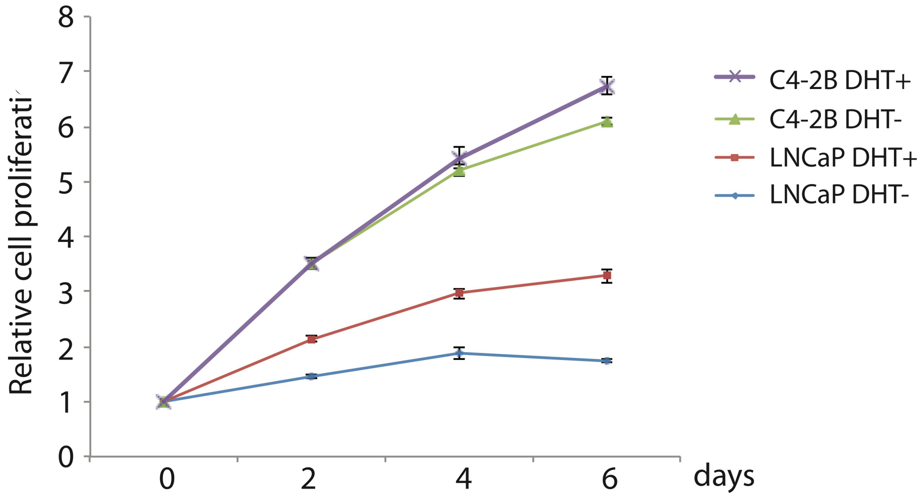

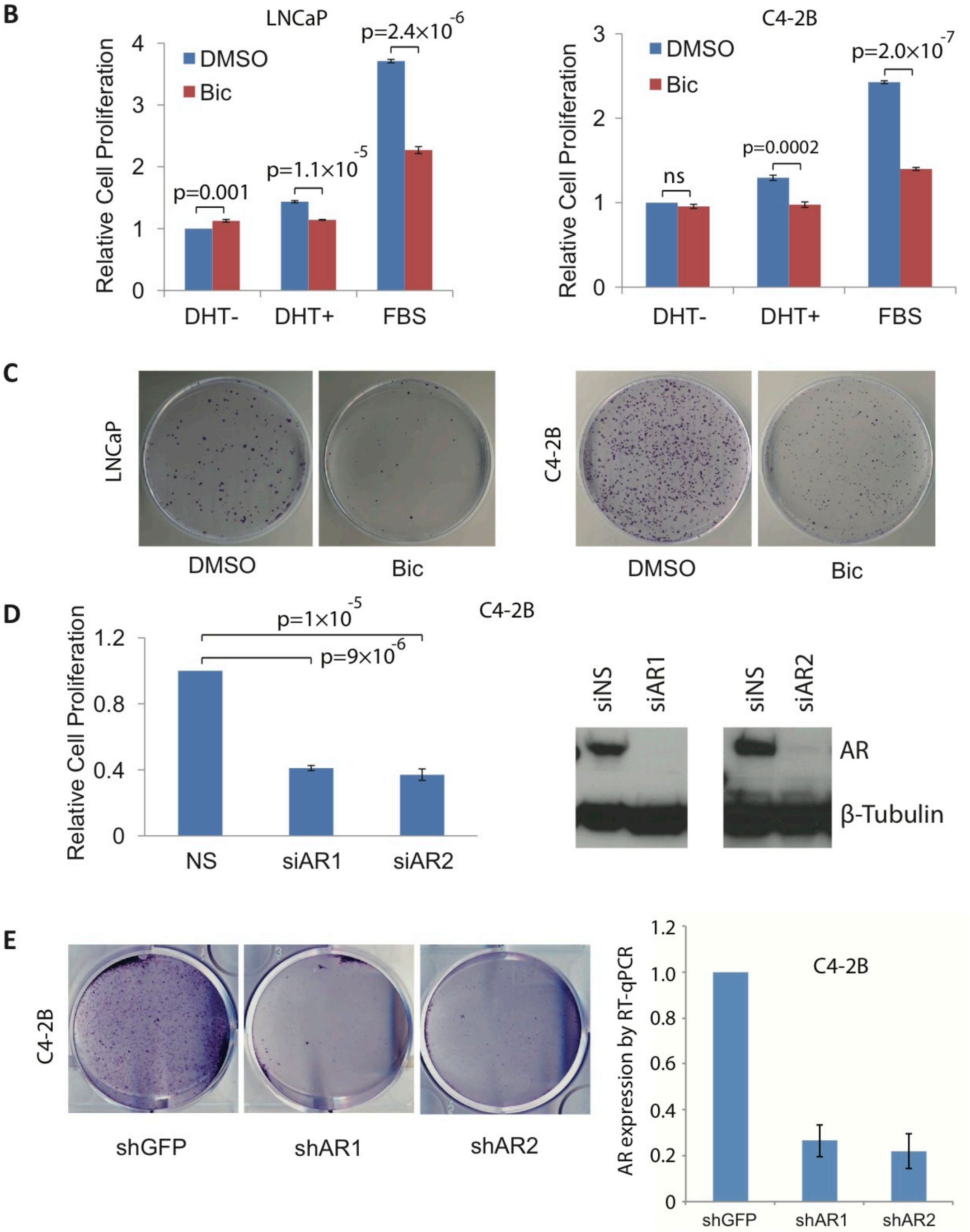

B
LNCaP
Relative Cell Proliferation
DMSO
Bic
p=0.001
p=1.1×10-5
p=2.4×10-6
DHT-
DHT+
FBS
C4-2B
Relative Cell Proliferation
DMSO
Bic
ns
p=0.0002
p=2.0×10-7
DHT-
DHT+
FBS
C
LNCaP
DMSO
Bic
C4-2B
DMSO
Bic
D
C4-2B
Relative Cell Proliferation
p=1×10-5
p=9×10-6
NS
siAR1
siAR2
siNS
siAR1
siNS
siAR2
AR
β-Tubulin
E
C4-2B
shGFP
shAR1
shAR2
AR expression by RT-qPCR
C4-2B
shGFP
shAR1
shAR2

Figure S2. Validation of AD-ORs and AI-ORs in LNCaP and C4-2B cells by ChIP-qPCR. LNCaP and C4-2B cells were grown in phenol red-free RPMI 1640 containing 5% charcoal-stripped serum (CSS) for 3 days and then treated with ethanol or DHT (10 nM) for 4 h (for LNCaP) or 16h (for C4-2B). Conventional site-specific ChIP assays were performed with anti-AR antibody. Seven AD-ORs and seven AI-ORs were analyzed by TaqMan or SYBR real-time PCR. The enrichment of AR occupancy was normalized to a negative control region (CTL). AD-OR1 is the PSA enhancer. AI-ORs1, 2, and 10 are located at promoter regions. Real-time PCR primer and probe sequences are listed in Supplementary File S1.

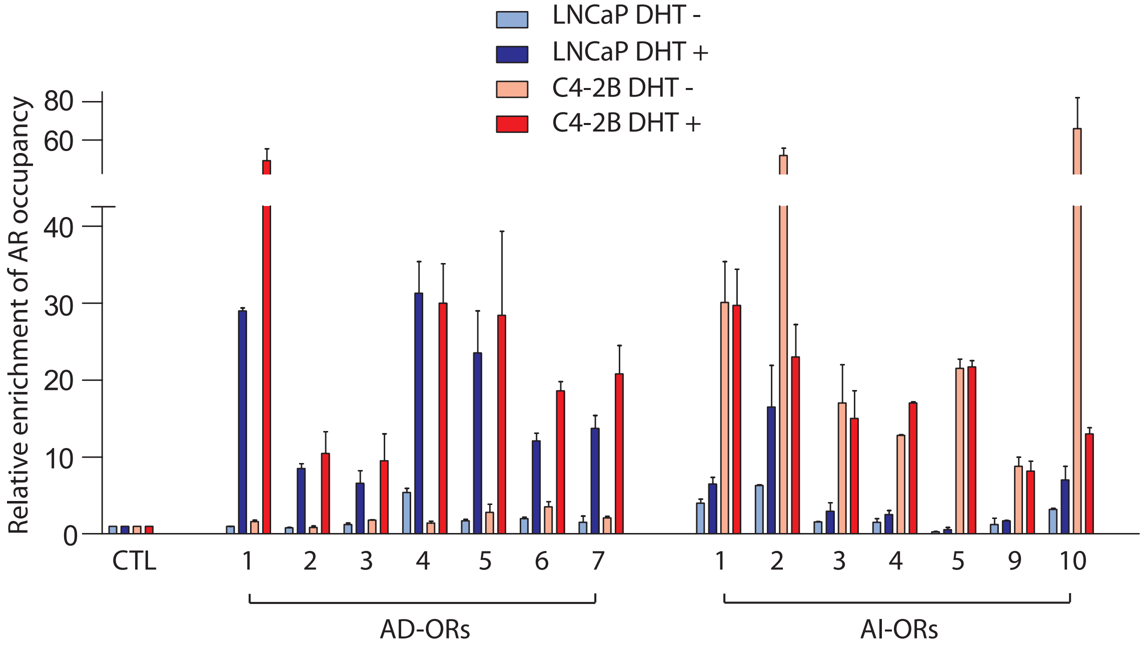

Figure S3. Androgen-independent AR binding is observed in CRPC 22RV1 cells. (A) 22Rv1 cells (from ATCC) were cultured in phenol red-free RPMI 1640 media supplemented with 5% charcoal-stripped serum for 3 days. After treatment with ethanol or DHT (10 nM) for additional 4 h, ChIP-seq experiments were performed as described in the Method section. *left* ChIP-seq signal intensity in a ± 2kb window for AR peaks called in 22RV1 DHT- and 22RV1 DHT+ cells using SISSRS (p-value cutoff = 0.01). 22RV1 AR sites overlapping AD and AI-ORs are marked in red and green, respectively. *right* Venn diagram showing overlap of 22RV1 DHT- and 22RV1 DHT+ AR peaks with AD and AI-ORs. **(B)** Representative AD-ORs and AI-ORs in 22RV1 and C4-2B cells.

A.

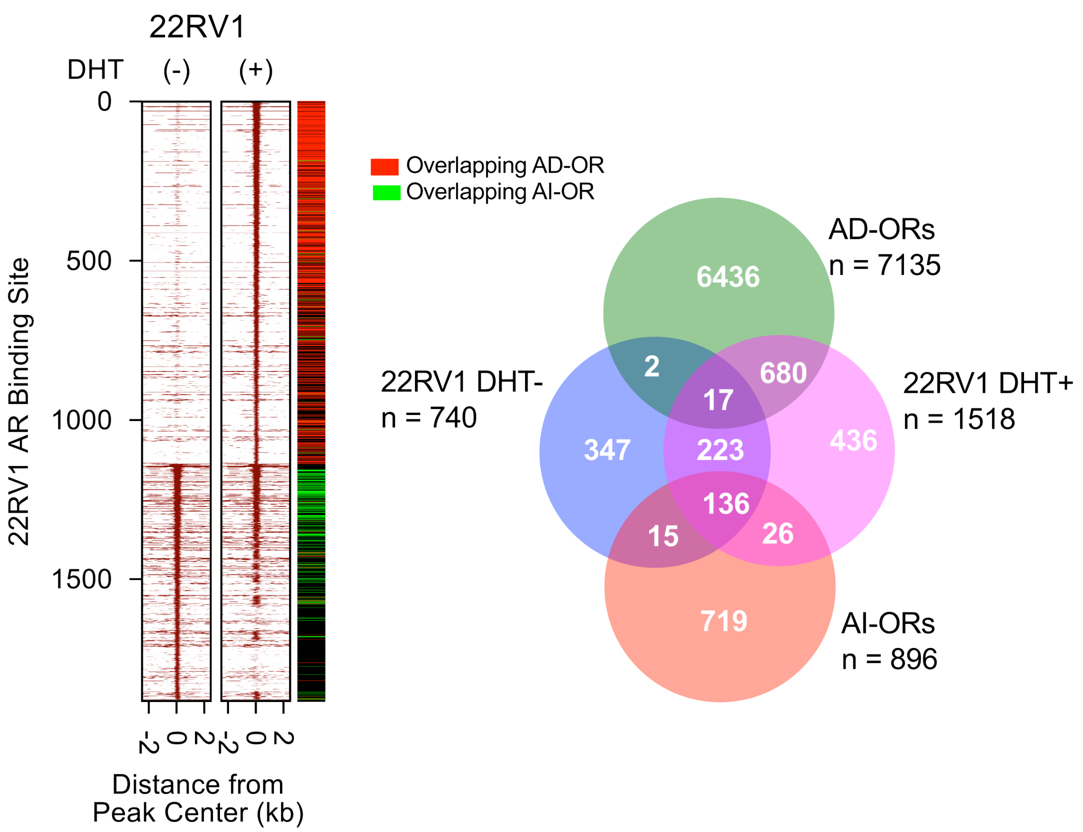

B

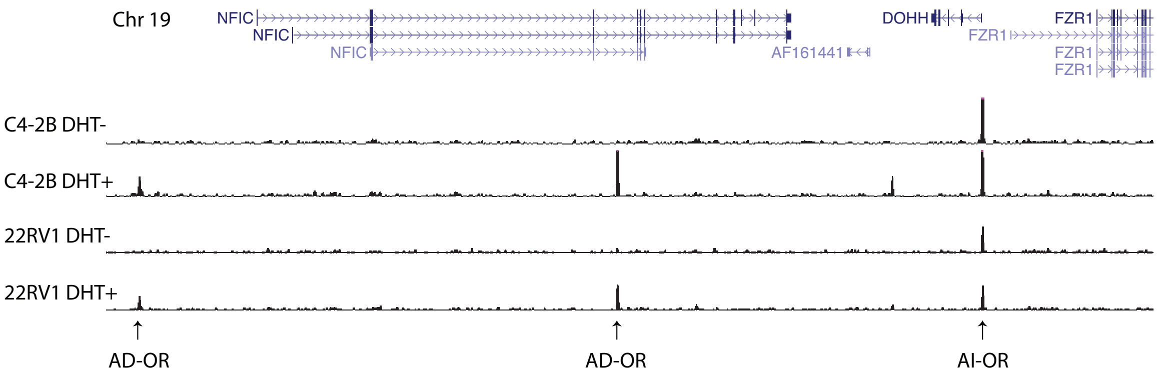

Figure S4. Sequence motifs at AD-ORs and AI-ORs. (A) Top two motifs discovered at repeat masked AD-OR sequences using MEME (http://meme.nbcr.net). Discovered motifs matched canonical AR (*top*) and FoxA1 (*bottom*) motifs. The AR motif is also known as androgen response element (ARE). **(B)** Results of motif discovery at C4-2B AI-OR sequences using MEME. *De novo* motif search using repeat masked AI-OR sequences located at proximal promoters led to discovery of a novel AI-OR promoter motif (*left*). This novel motif does not match any known motifs in the JASPAR, TRANSFAC and UNIPROBE databases based on motif comparison using TOMTOM (1). The top two overrepresented motifs discovered using AI-OR sequences located at tRNA (without repeat masking) matched the tRNA A-box and tRNA B-box. **(C)** Results of AME search for the AR, FoxA1, AI-OR promoter, A-box and B-box motifs discovered using MEME. Motif search was performed using repeat masked sequences for 7,135 AD-ORs, 276 AI-OR bound promoters and 538 non-tRNA/non-promoter AI-ORs and using non-repeat masked sequences for 54 AI-OR bound tRNAs. Asterisks mark statistically enriched motifs as determined by AME. For AD-ORs and non-tRNA/non-promoter AI-ORs, enrichment was determined relative to shuffled sequences with matched dinucleotide content. For AI-OR bound promoters, enrichment was determined relative to unbound high CpG (HCG) content promoters, with CpG content computed as described in Saxonov et al (2). Note that 284 AI-ORs mapped to 276 promoters because some promoters were bound by multiple AI-ORs. For tRNAs, enrichment was determined relative to unbound tRNA in the tRNA database. Note that 20 tRNAs annotated in repeat masker 3.2.7 but not included in the tRNA database were excluded from AME analysis.

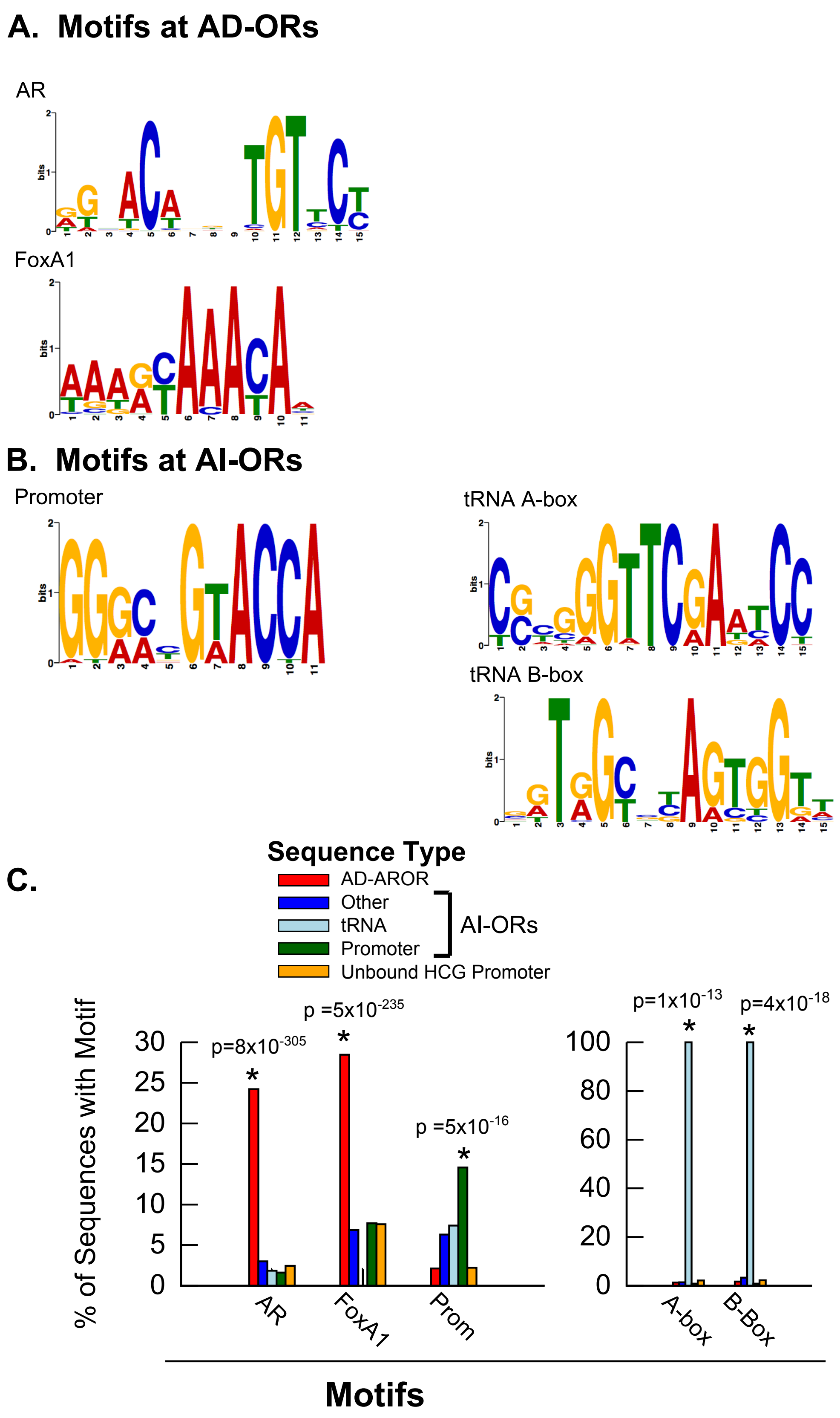
A. Motifs at AD-ORs
AR
FoxA1
B. Motifs at AI-ORs
Promoter
tRNA A-box
tRNA B-box
C.
Sequence Type
AD-AROR
Other
tRNA
Promoter
Unbound HCG Promoter
AI-ORs
% of Sequences with Motif
p=8x10-305
p =5x10-235
p =5x10-16
p=1x10-13
p=4x10-18
AR
FoxA1
Prom
A-box
B-Box
Motifs

Figure S5. FoxA1 is enriched at AD-ORs but not AI-ORs. FoxA1 ChIP-seq signal intensity in a ± 2kb window at AR binding sites in C4-2B cells. AD and AI-ORs are marked in red and green, respectively

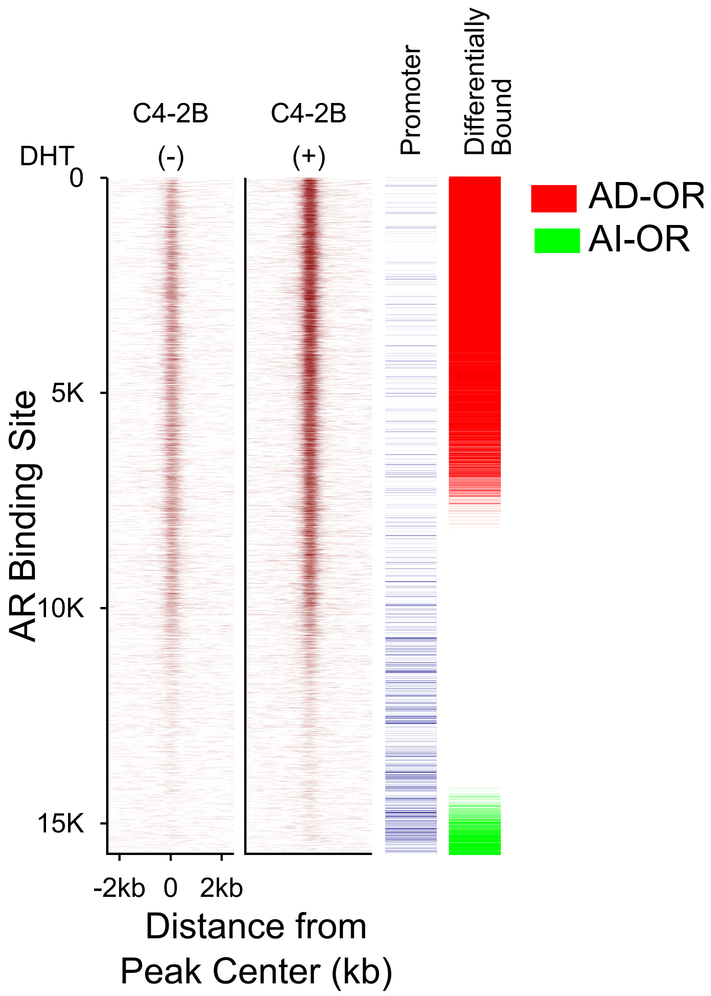

Figure S6. Characteristics of AR-bound promoters.

(A) Histogram of distance relative to the transcription start site (TSS) of the nearest gene for 284 promoter AI-ORs. Promoter AI-ORs are frequently located immediately upstream of the TSS. **(B)** Frequency histograms of normalized CpG content for 276 AI-OR bound promoters and 31,999 promoters based on ensembl gene annotations. AI-OR bound promoters have high CpG content. Note that the number of AI-OR bound promoters differs from the number of AI-ORs at promoters because some promoters are bound by multiple AI-ORs.

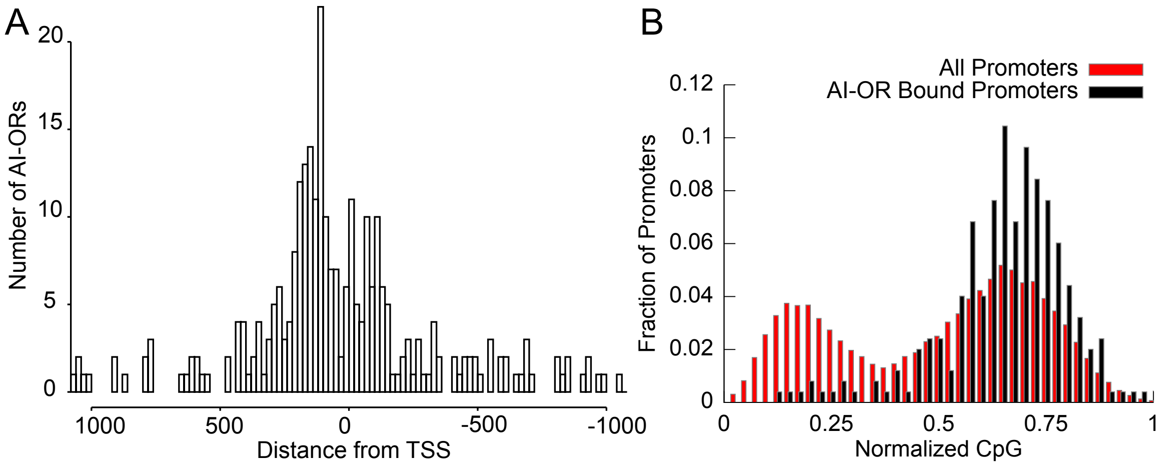

Figure S7. AR is knocked down by AR siRNA. (A) AR knockdown validated by Western blots. C4-2B cells were transfected with AR siRNA (siAR) or non-specific siRNA (siNS) using the Forward and Reverse Transfection Protocols (Invitrogen) in the experiment 1 and 2 respectively. After transfection, cells were grown in phenol red-free RPMI 1640 containing 5% CSS for 72 h prior to RNA-seq analyses. **(B)** Inhibition of AR transcription was also confirmed by RNA-seq results.

A.

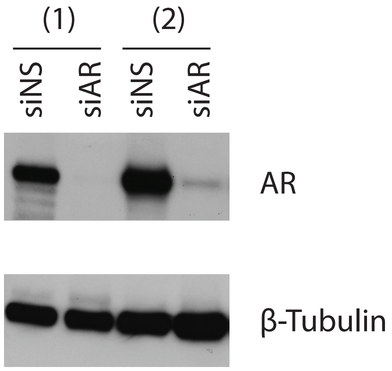

B.

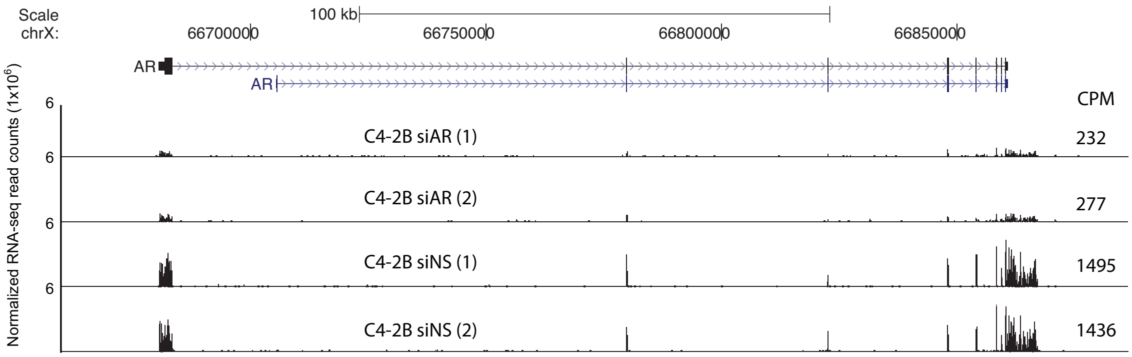

Figure S8. Expression of androgen-independent (AI) regulated genes does not correlate with proximal promoter binding. % of genes with an AI-OR at the proximal promoter for all genes, DHT regulated and AI regulated genes. No significant (N.S.) correlation between promoter AI-ORs and expression of proximal genes was observed.

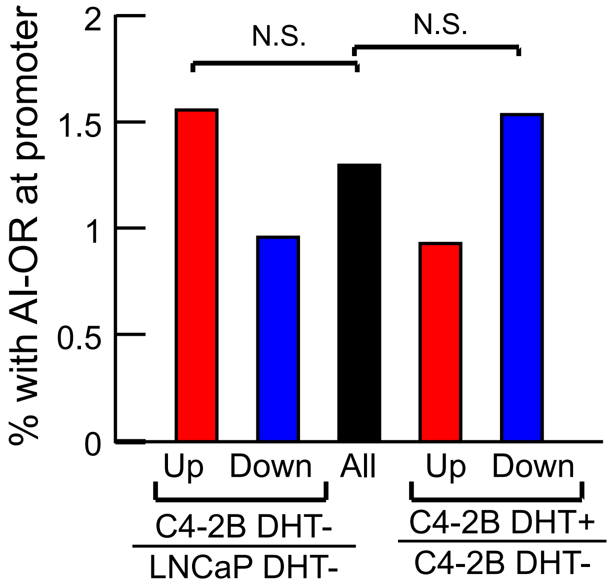

Figure S9. AI- upregulated genes are over-represented in DHT down- and siAR down-regulated genes. % of All genes and AI-upregulated genes knocked down by siAR **(A)** and downregulated by DHT **(B).** Statistical significance was determined by a hypergeometric test.

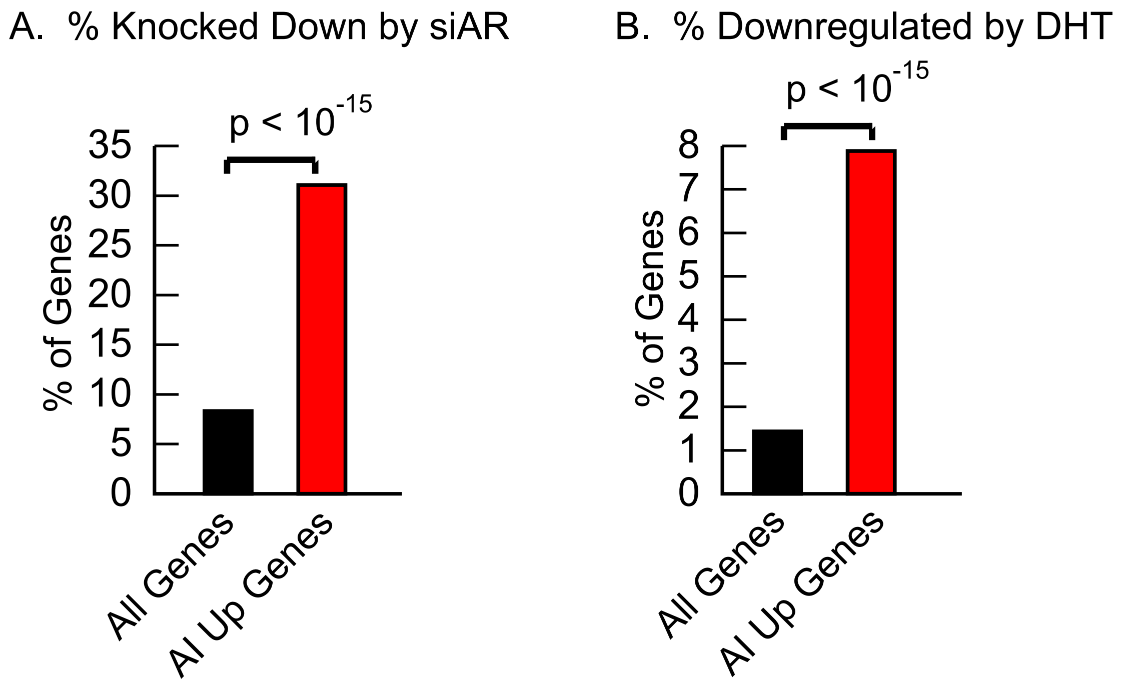

Figure S10. Interaction of AI-ORs and AI-upregulated genes confirmed by 3C. (A) 3C-qPCR shows increased interaction between the AI-OR at the DGAT2 promoter and the SERPINH1 promoter compared to nearby regions in C4-2B cells. While the AI-OR is located at the DGAT2 promoter, SERPINH1 is the nearest AI-upregulated gene (11.6-fold induction, p=2.69E-13) in this region. 3C assays were performed using EcoRI in LNCaP and C4-2B cells in the absence of DHT (see the Method section). Small black arrows represent the primers and a short red line indicates the probe for 3C-qPCR. The results are presented as the average ± standard deviation of two independent 3C libraries. **(B)** 3C-qPCR shows strong interaction between the AI-OR and the SDC1 promoter in C4-2B cells. The non-promoter/non-tRNA AI-OR is located 127 kb away from the AI-upregulated gene SDC1 (2.1-fold induction, p=0.0021). 3C assays were performed using BamHI in LNCaP and C4-2B cells in the absence of DHT. Gene expression of SERPINH1 and SDC1 in LNCaP and C4-2B cells were measured by RNA-seq (provided in Supplementary File 3).

A.

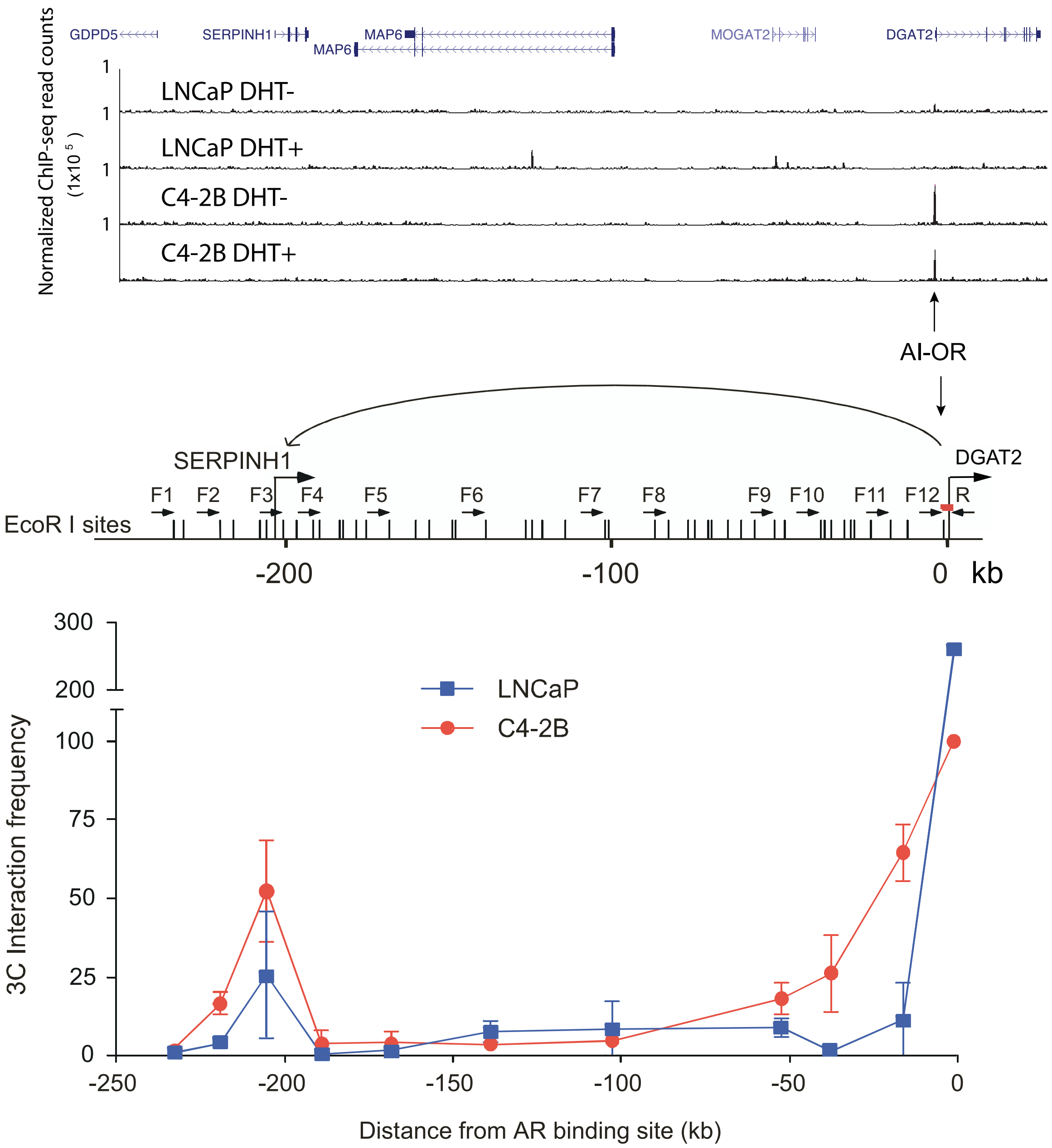

GDPD5
SERPINH1
MAP6
MAP6
MOGAT2
DGAT2
Normalized ChIP-seq read counts (1x10^5)
LNCaP DHT-
LNCaP DHT+
C4-2B DHT-
C4-2B DHT+
AI-OR
SERPINH1
DGAT2
F1 F2 F3 F4 F5 F6 F7 F8 F9 F10 F11 F12 R
EcoR I sites
-200 -100 0 kb
LNCaP
C4-2B
3C Interaction frequency
Distance from AR binding site (kb)

B.

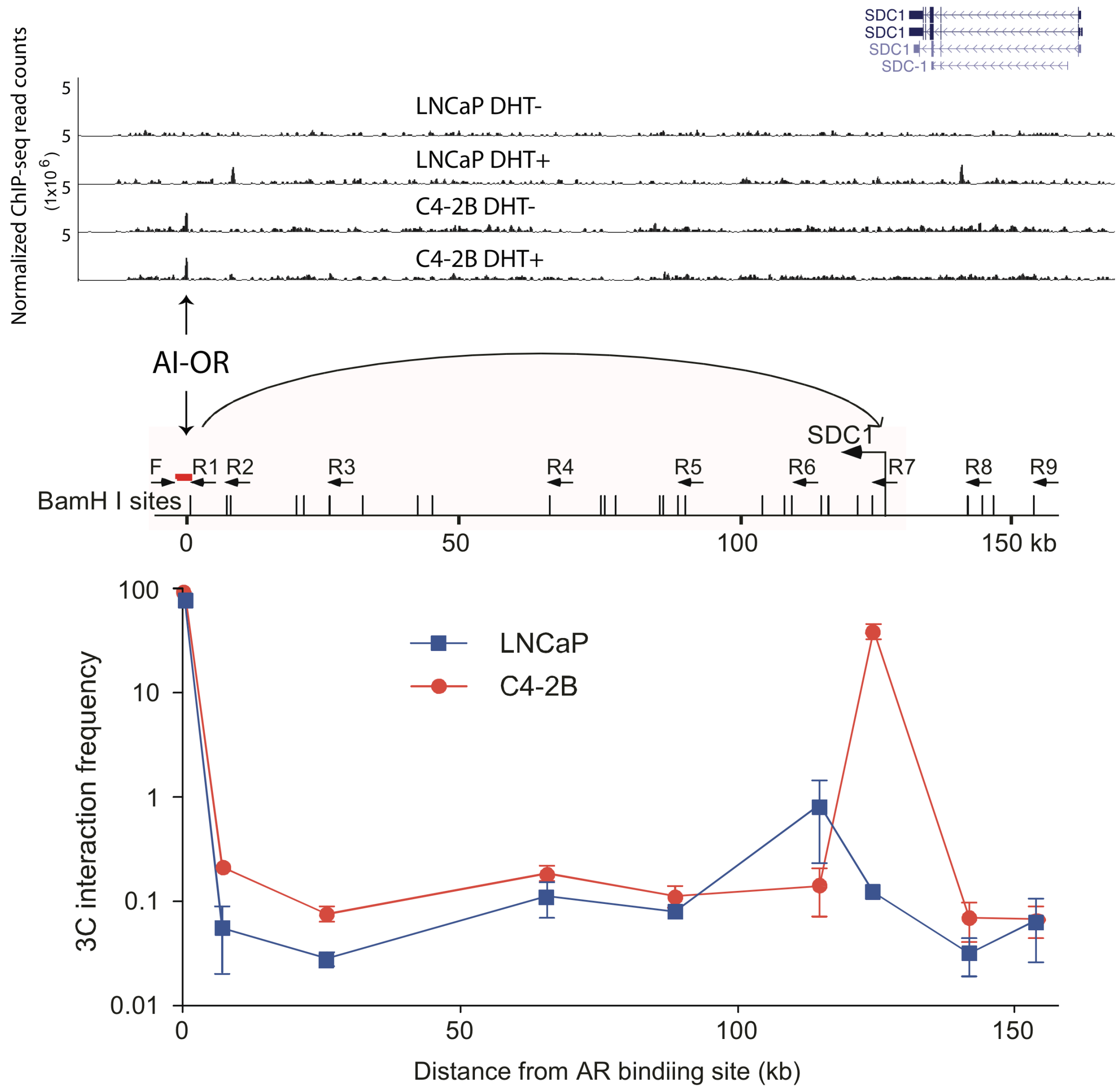
SDC1
SDC1
SDC1
SDC-1
Normalized ChIP-seq read counts (1x10^6)
5
LNCaP DHT-
5
LNCaP DHT+
5
C4-2B DHT-
5
C4-2B DHT+
AI-OR
SDC1
F
R1
R2
R3
R4
R5
R6
R7
R8
R9
BamH I sites
0
50
100
150 kb
3C interaction frequency
100
10
1
0.1
0.01
LNCaP
C4-2B
0
50
100
150
Distance from AR bindiing site (kb)

Figure S11. AI upregulated genes are associated with cell cycle related functions. Statistically significant map folders (FDR = 0.05) for AI upregulated (top) and DHT upregulated genes as determined using Metacore by GeneGo Inc.

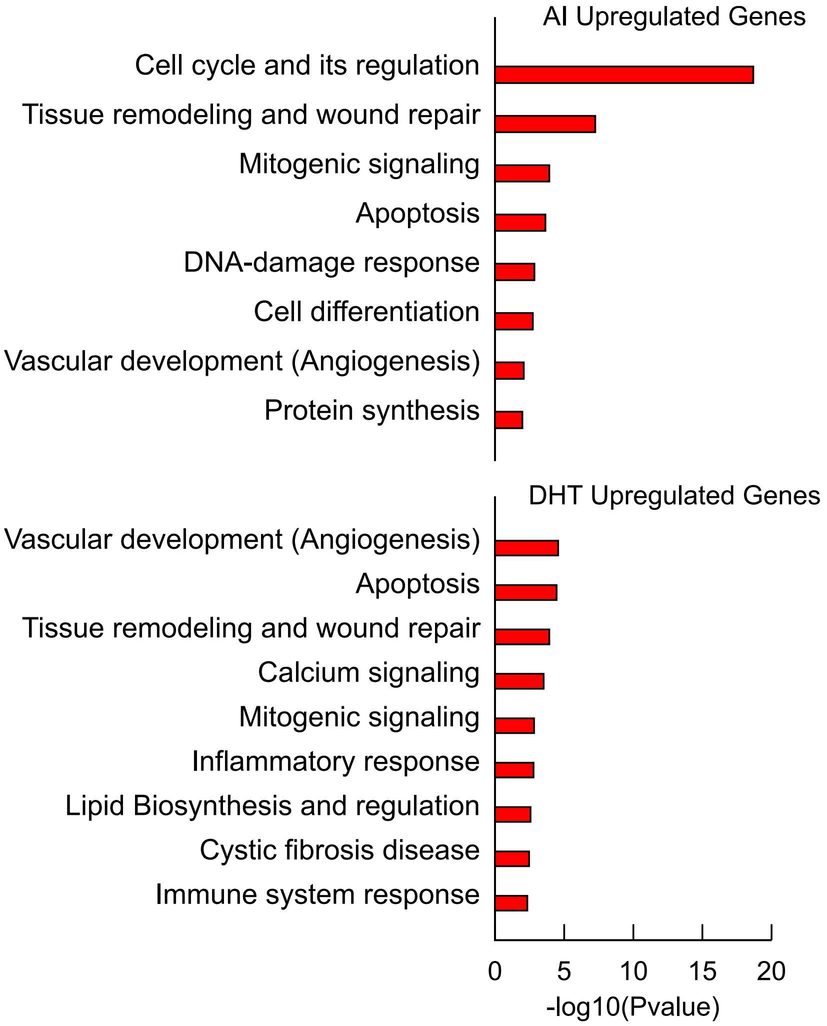

Figure S12. LNCaP-abl and C4-2B models share common basal androgen-independent upregulated genes but have distinct AR binding sites (A) % of All, C4-2B DHT-/LNCaP DHT- Up, C4-2B DHT-/LNCaP DHT- Up "cell cycle phase", and C4-2B DHT-/LNCaP DHT- down genes that are also LNCaP-abl DHT-/LNCaP DHT- upregulated. Microarray data was obtained from GSE11428 and GSE7868 and differentially expressed genes were called using limma (3) at FDR = 0.05 and Fold Change > 1.5. **(B)** % of AD and AI-ORs overlapping with AR ChIP-chip peaks in androgen-dependent (LNCaP) and androgen-independent (LNCaP-abl) cell lines at FDR < 0.01. ChIP-ChIP data was downloaded from the Brown lab website. (http://research4.dfci.harvard.edu/brownlab/datasets/index.php).

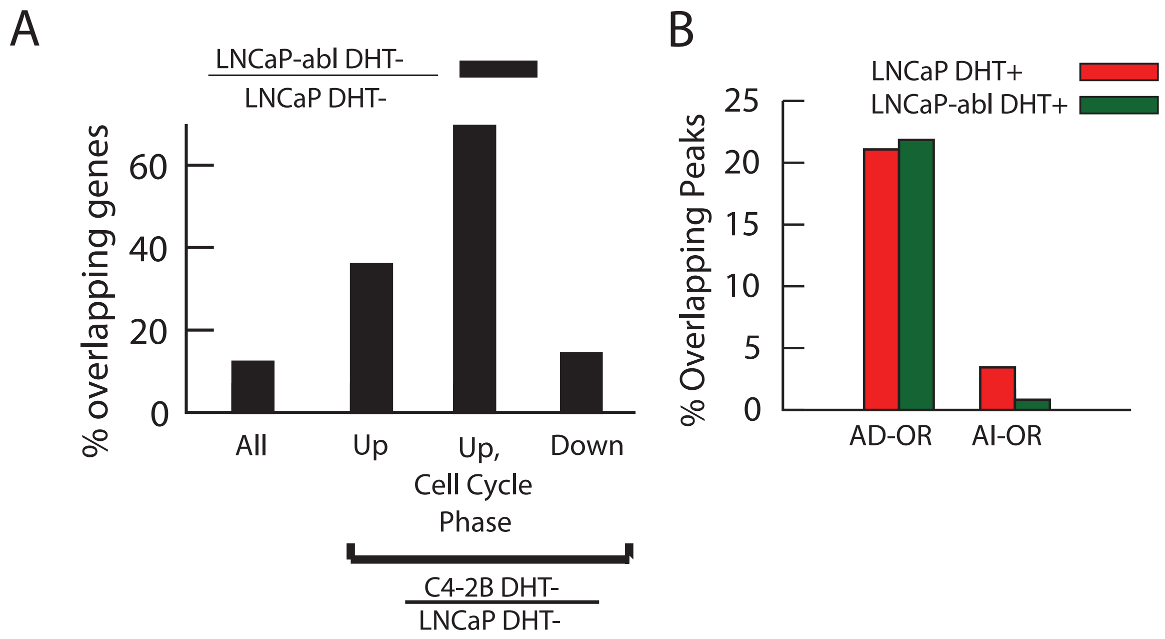

REFERENCES

1. Gupta, S., Stamatoyannopoulos, J.A., Bailey, T.L. and Noble, W.S. (2007) Quantifying similarity between motifs. *Genome Biol*, **8**, R24.
2. Saxonov, S., Berg, P. and Brutlag, D.L. (2006) A genome-wide analysis of CpG dinucleotides in the human genome distinguishes two distinct classes of promoters. *Proc Natl Acad Sci U S A*, **103**, 1412-1417.
3. Smyth, G.K. (2004) Linear models and empirical bayes methods for assessing differential expression in microarray experiments. *Stat Appl Genet Mol Biol*, **3**, Article3.
